# Supplementary material for: Structure and unusual binding mechanism of the hyaluronan receptor LYVE-1 mediating leucocyte entry to lymphatics
Source: Nat Commun. 2025 Mar 20;16:2754. doi: 10.1038/s41467-025-57866-8 (PMC11926218; doi:10.1038/s41467-025-57866-8)
Supplement: Supplementary file 1 — Supplementary Information [file 41467_2025_57866_MOESM1_ESM.pdf]

SUPPLEMENTARY INFORMATION

This file includes: Supplementary Figures 1 to 21 and Supplementary Tables 1 to 4

Supplementary Figures

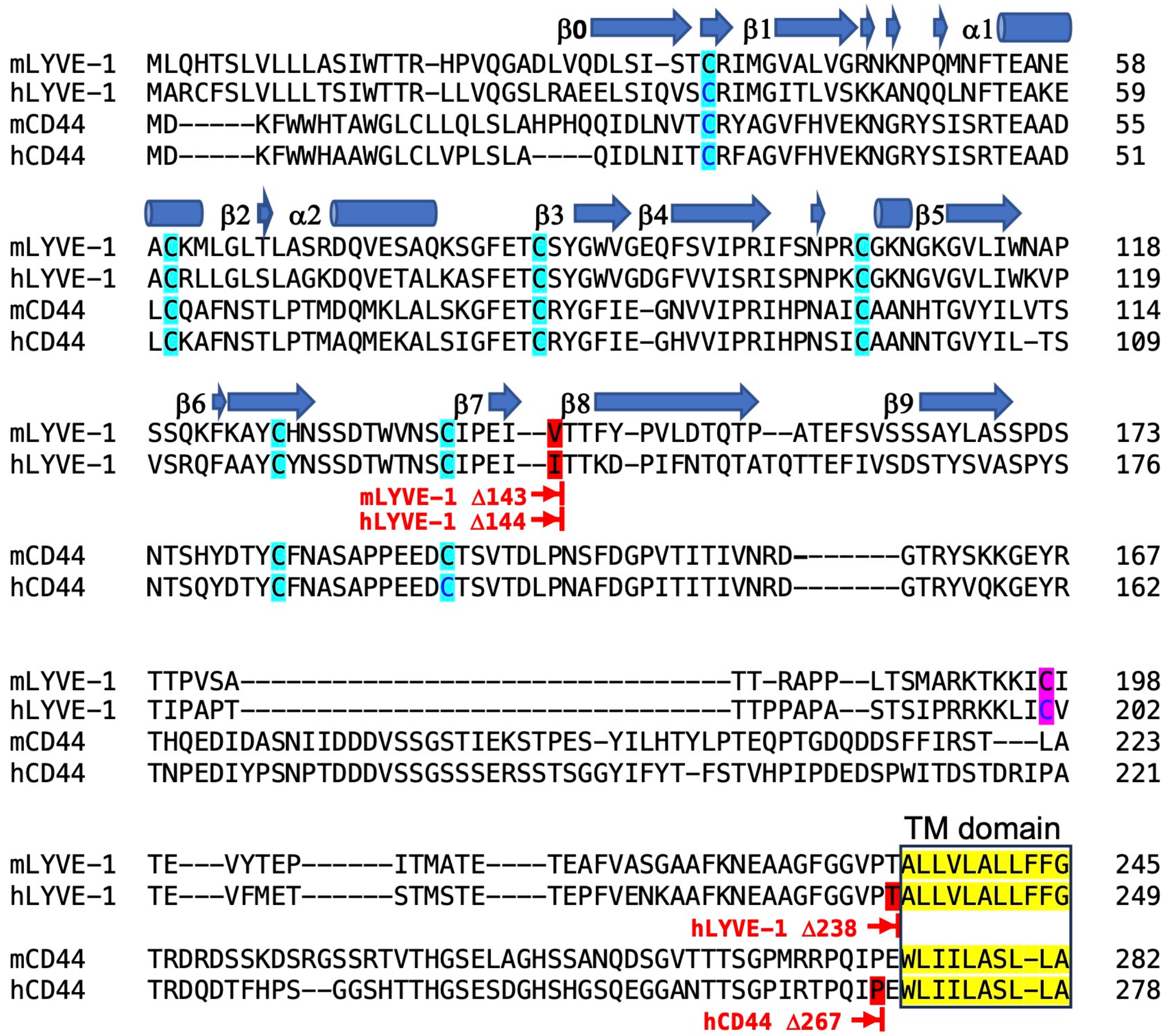

Supplementary Figure 1. LYVE-1 and CD44 ectodomain sequence alignments showing boundaries of constructs used for crystallography and DFS. Mouse (m) and human (h) LYVE-1 and CD44 amino acid sequences (NP_444477.2, NP_006682.2, NP_001034240.1 and NP_001001391.1 respectively) aligned using Clustal Omega [1], highlighting key conserved cysteine residues (turquoise) and secondary structure elements (blue) of the Link module as determined from the crystal structures in each case. Note the β8 and β9 strands in CD44 appear not to be conserved in LYVE-1 where the equivalent region is predicted to be unstructured. The additional unpaired cysteine in LYVE-1 that mediates homodimer formation is highlighted in pink and the initial region of the transmembrane domain in all four sequences in yellow. The soluble LYVE-1 and CD44 HA-binding domain constructs used for crystallography and the longer soluble full ectodomain constructs (including the non-dimerising LYVE-1 Cys201Ala mutant) used for DFS were truncated as indicated in red type.

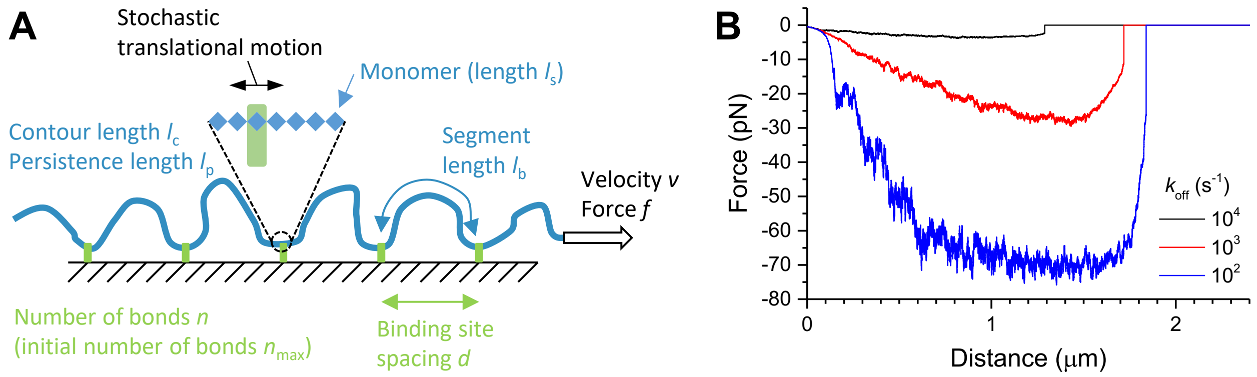

Supplementary Figure 2. **Reductionist model for dynamic force spectroscopy (DFS) of sliding bonds.** (A) Schematic illustration of the reductionist computational model. A polymer chain (with contour length l_c, persistence length l_p and monomer length l_s; blue) bound to a number n of surface-anchored receptors (spaced apart by the distance d; green) thus forming n +1 segments (*i.e.*, two tails and n – 1 loops) of contour length l_b is pulled at a constant velocity v. Forces act on each bond owing to the entropic worm-like chain elasticity of the adjacent chain segments. Translational motion of the chain along a given receptor is implemented by stochastic bond rupture followed by reformation *via* a monomer that is adjacent (left or right) to the one from which rupture occurred; the chain permanently detaches from a given receptor when the chain end is reached. At zero net force, the rate of bond rupture is k_{off}; forces modify the free energy landscape, enhancing the bond breakage rate in the direction of (net) force and reducing it in the opposite direction, as defined by the Bell-Evans theory (with the bond length $x_\beta = l_s/2$ defined by symmetry constraints).
Model parameters were matched to the experimental conditions used to probe hLYVE-1•HA interactions by DFS: l_c = 2.1 µm (for HA-b 840), l_p = 4 nm, l_s = 1.0 nm (equal to the contour length of a GlcUA-GlcNAc disaccharide, the chemical monomer of HA), v = 1 µm·s^{-1}; d = 50 nm and initial bond number n_{max} = 9 are rough approximations based on the estimated surface coverage of LYVE-1 and the reach of an HA chain with little diversion from its random coil morphology (radius of gyration 75 nm for HA-b 840) [2]; $l_b = l_c / (n_{max} + 1)$ = 210 nm. For simplicity, we ignore the stochastic variations in d and initial l_b values that would be expected in the experiment.
(B) Representative force *vs.* distance curves obtained with the reductionist model using bond rupture rates k_{off} between 10^2 and 10^4 s^{-1} (colour coded as indicated in the graph). Data are representative of three repeats of the simulation process with identical starting conditions. The gross shape of the curves is similar across all k_{off} values, and comparable to the experimental force curves (Figure 1C). The magnitude of the force, and the distance at which the HA chain fully detaches from the surface, depend sensitively on the off rate. Best correspondence with experimental data is obtained for $k_{off} \approx 10^3$ s^{-1}. This reductionist model demonstrates that such high translation rates entail effective sliding of the HA chain along LYVE-1. Source data are provided as a Source Data file.

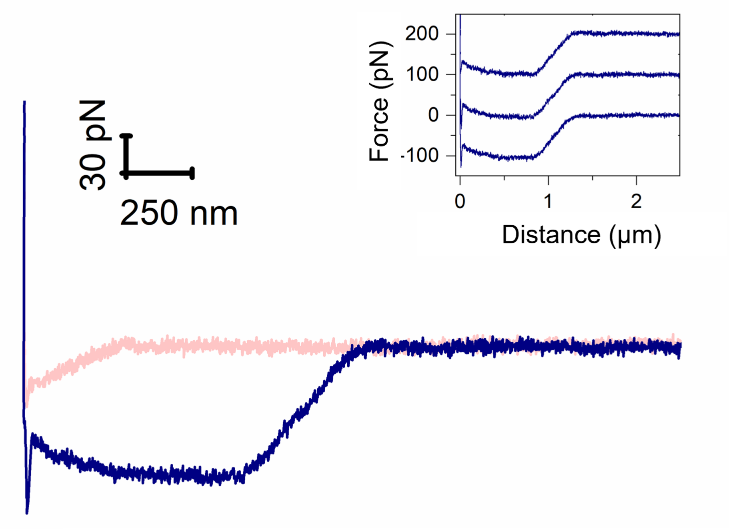

Supplementary Figure 3. The nature of LYVE-1•HA interactions is preserved for LYVE-1 monomers. Representative force *vs.* distance curve obtained by DFS with the LYVE-1 Cys201Ala mutant which forms exclusively monomers. The shape of the curve is qualitatively similar to Main Figure 1C, indicating that the distinctive nanomechanical property of LYVE-1•HA bonds persists when LYVE-1 is presented in its monomeric form. The *inset* shows three randomly selected retract curves (from a total of 100 curves; with *y* axes offset for clarity), and their similarity confirms the deterministic nature of the interaction as also seen in Main Figure 1C. Assay conditions: HA-b 840 grafted to AFM probe and hLYVE-1 Cys201Ala Δ238 with a His_{10} single tag anchored on His-tag-capturing PEG; hLYVE-1 Cys201Ala Δ238 His_{10} incubation concentration: 5 µg/ml; approach/retract velocity: 1 µm/s. Data are representative of three independent experiments with distinct HA-coated probes and receptor-coated substrates. Source data are provided as a Source Data file.

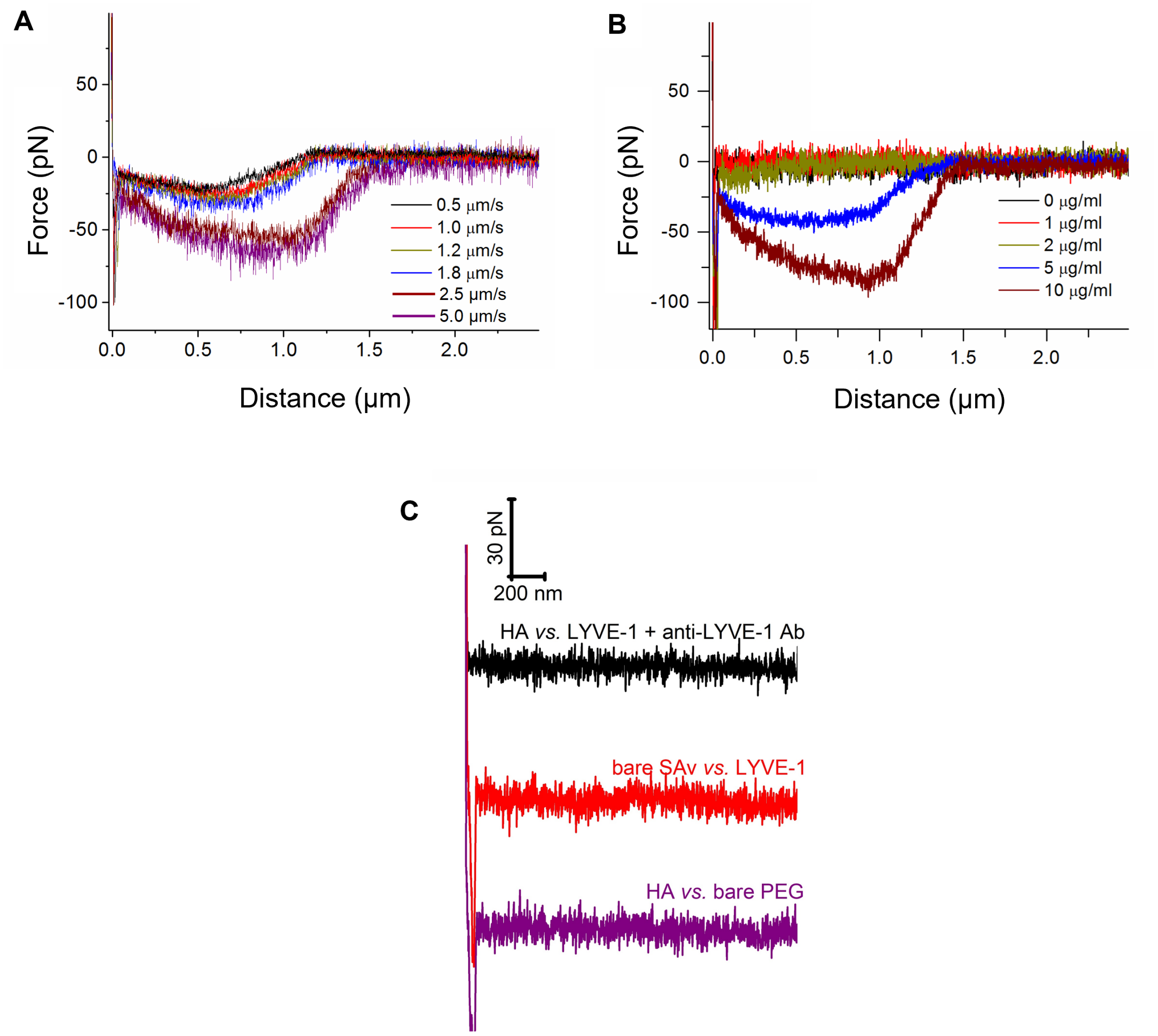

Supplementary Figure 4. Effect of pulling velocity and receptor surface coverage on LYVE-1•HA interactions. (A) Representative force *vs.* distance curves recorded with HA grafted *via* the reducing end at different retract velocities (colour coded as indicated). The magnitude of forces and the interaction range increase with velocity *v*. Assay condition: HA-b 840 grafted to AFM probe and hLYVE-1 Δ238 with a His_{10} single tag anchored on His-tag-capturing PEG; hLYVE-1 Δ238 His_{10} incubation concentration: 5 µg/ml. (B) Representative force *vs.* distance curves recorded with HA grafted *via* the reducing end at different LYVE-1 densities (incubation concentrations from 0 to 10 µg/ml are colour coded and cover the range from zero to maximal LYVE-1 surface density). Forces are below the detection limit up to 1 µg/ml and then increase with LYVE-1 surface density. Assay condition: HA-b 840 grafted to AFM probe and hLYVE-1 Δ238 with a His_{10} single tag anchored on His-tag-capturing PEG; approach/retract velocity: 1 µm/s. (C) Representative force vs. distance curves recorded for control conditions as indicated: HA binding was impaired by a function-blocking anti-LYVE-1 antibody (HA *vs.* LYVE-1 + anti-LYVE-1 Ab), or either HA-b (bare SAv *vs.* LYVE-1) or LYVE-1 (HA *vs.* bare PEG) were omitted in the interaction assay. No interaction forces were observed beyond 100 nm tip-substrate distance, and this feature was representative for all force curves registered for the control conditions (n = 200 each). Basic assay conditions: HA-b 840 grafted to AFM probe and hLYVE-1 Δ238 with a His_{10} single tag anchored on His-tag-capturing PEG, approach/retract velocity 2 µm/s. Data are representative of two independent experiments with distinct probes and substrates per condition. Source data are provided as a Source Data file.

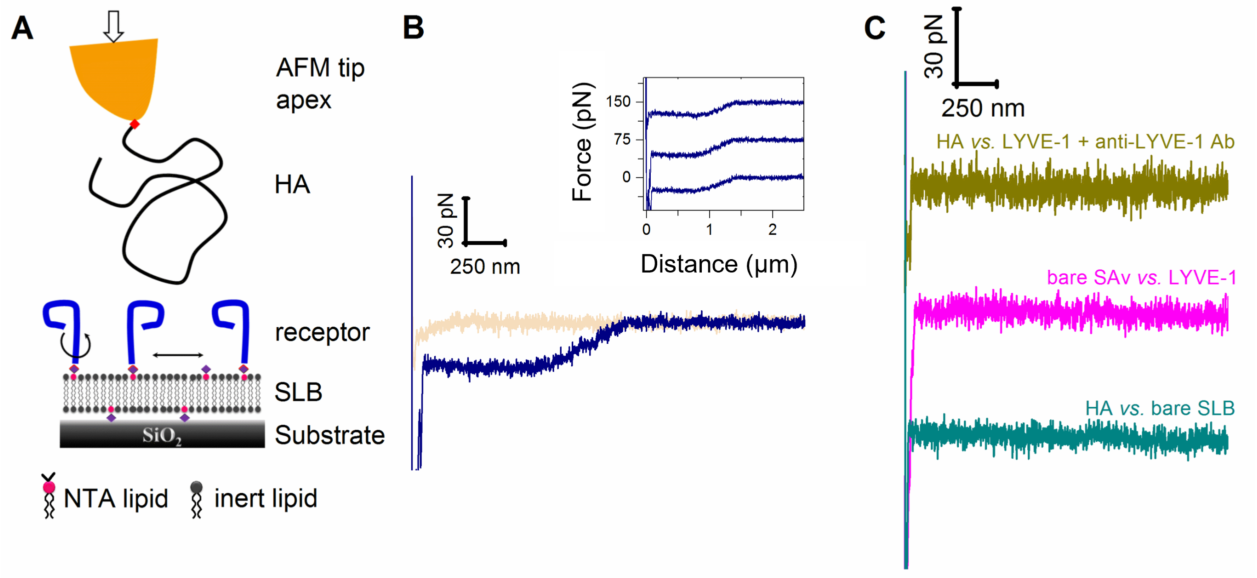

Supplementary Figure 5. The lateral mobility of LYVE-1 does not affect the nature of LYVE-1•HA interactions. (A) Schematic illustration of the modified DFS interaction assay. Anchorage of LYVE-1 to a fluid supported lipid bilayer (SLB) affords lateral receptor mobility, in contrast to PEG (Main Figure 1) where the receptors are immobile. HA is grafted via the reducing end to the AFM tip. (B) Representative force *vs.* distance curve obtained with SLB-anchored LYVE-1. The shape of the curve is qualitatively similar to Figure 1C, indicating that the distinctive nanomechanical property of LYVE-1•HA bonds persists also when LYVE-1 is laterally mobile. The inset shows three randomly selected retract curves (from a total of 100 curves; with *y* axes offset for clarity), and their similarity confirms the deterministic nature of the interaction as also seen in Figure 1C. (C) Representative force *vs.* distance curves recorded for control conditions as indicated: HA binding was impaired by a function-blocking anti-LYVE-1 antibody (HA vs. LYVE-1 + anti-LYVE-1 Ab), or when either HA-b (bare SAv vs. LYVE-1) or LYVE-1 (HA vs. bare SLB) were omitted in the interaction assay. No interaction forces were observed beyond 100 nm tip-substrate distance, and this feature was representative for all force curves registered for the control conditions (n = 200 each). Basic assay conditions: HA-b 840 grafted to AFM probe and hLYVE-1 Δ238 with a His_{10} single tag anchored on His-tag-capturing SLBs; approach/retract velocities: 1 µm/s in (B) and 2 µm/s in (C). Data are representative of two independent experiments with distinct probes and substrates per condition. Source data are provided as a Source Data file.

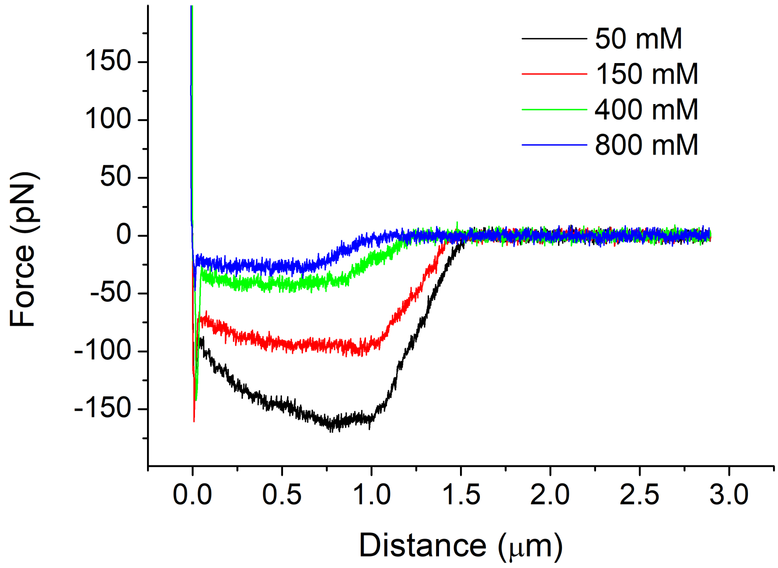

Supplementary Figure 6. The salt concentration affects the magnitude of interaction forces but not the nature of LYVE-1•HA interactions. Representative force *vs.* distance curves (retract only) are shown for different NaCl concentrations (between 50 and 800 mM, as listed with colour codes), all obtained with the same HA-coated AFM probe and LYVE-1-coated substrate (data were randomly selected from a total of 20 curves per condition). The shape of all curves is qualitatively similar, indicating that the distinctive nanomechanical property of LYVE-1•HA bonds persists, irrespective of the ionic strength. However, the magnitude of the forces decreases with ionic strength confirming that electrostatic interactions make a substantial contribution to LYVE-1•HA bonds as already previously shown with binding assays in the absence of force [2]. Assay conditions: HA-b 840 grafted to AFM probe and hLYVE-1 Δ238 with a His_{10} single tag anchored on His-tag-capturing PEG, approach/retract velocity: 1 μm/s. Data are representative of one HA-coated probe and four independent receptor-coated substrates. Source data are provided as a Source Data file.

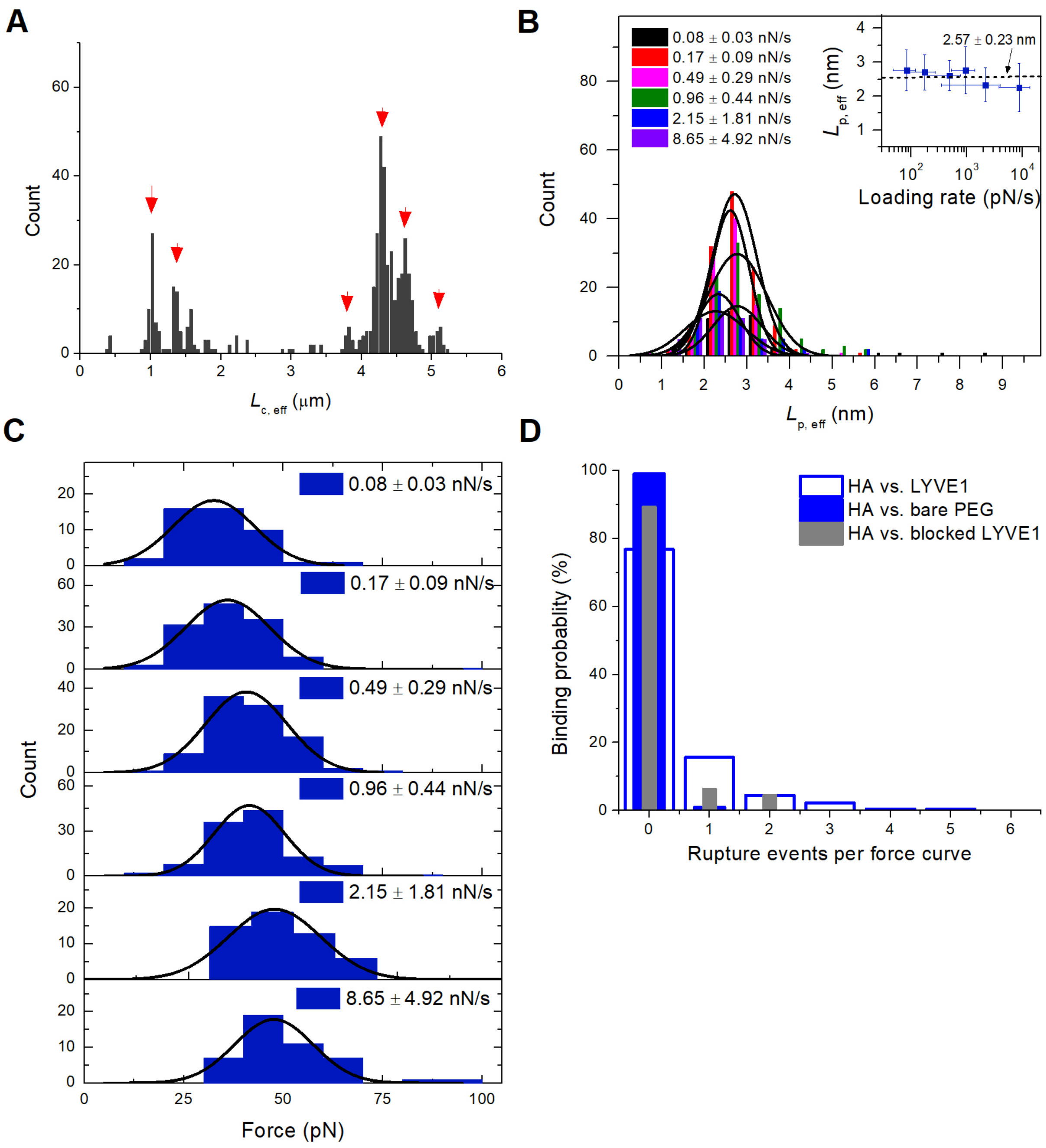

Supplementary Figure 7. Statistical analysis of LYVE-1 unbinding from HA loops. All data were determined through WLC model fits for the measurement described in Figure 2A-C. Assay conditions: HA-*g*-b (1.6% nominal degree of biotinylation per disaccharide) bound to AFM probe, and hLYVE-1 Δ238 with a biotin/His_{10} dual tag anchored on His-tag-capturing PEG (incubated at a concentration of 1.0 μg/mL (A-C) or 0.37 μg/mL (D) for 15 min). All force curves were collected with a surface dwell time τ_{dwell} = 3 s and a maximal load of 600 pN. (A) Histograms of the effective contour lengths $L_{c,eff}$; all data are combined and reveal a few discrete peaks (red arrowheads) which we assign to HA loops of discrete sizes ($L_{c,eff} \approx L_{c,loop}/2$, see below). (B) Histograms of the effective persistence lengths $L_{p,eff}$ with best-fit Gaussian curves; data for each of the six probed retract velocities (0.5, 1, 2, 4, 8 and 12 μm/s) are shown separately, and colour coded as indicated with their corresponding mean instantaneous loading rates (r = 0.08 ± 0.03, 0.17 ± 0.09, 0.49 ± 0.29, 0.96 ± 0.44, 2.15 ± 1.81, and 8.65 ± 4.92 nN/s, respectively). The inset shows the distribution of $L_{p,eff}$ as a function of instantaneous loading rate (in semi-log presentation; mean ± SD); the dashed horizontal line represents the global mean and the associated number the mean ± SD of the individual mean values. (C) Rupture force histograms for the six instantaneous loading rates (listed as mean ± SD).

Solid lines represent Gaussian fits leading to the mean rupture forces shown in Figure 2C. (D) Probability of specific rupture events per force curve for the interactions as indicated in the plot (retract velocities: 2 μm/s – HA *vs.* LYVE-1, and HA *vs.* LYVE-1 blocked with anti-LYVE-1 blocking mAb, 1 μm/s – HA *vs.* surface lacking LYVE-1). Pre-incubation of LYVE-1 with an anti-LYVE-1 antibody known to block HA binding strongly reduces the frequency of interaction, and virtually no event is observed on surfaces that lack LYVE-1, indicating that binding is specific. The analyses in (D) are based on 783 (HA *vs.* LYVE-1), 335 (HA *vs.* LYVE-1 blocked with anti-LYVE-1 blocking Ab) and 206 (HA *vs.* surface lacking LYVE-1) force curves.
The interaction system of one HA loop being stretched is physically equivalent to two HA chains of equal contour length being stretched in parallel. The effective persistence length shown in (B) should thus be close to half the persistence length of HA ($L_{p,eff} \approx L_{p,HA}/2$). $L_{p,eff} = 2.5 \pm 0.4$ nm (mean ± S.D. from $n = 3$ independent experiments with distinct HA-coated probes and receptor-coated substrates) indeed matches quite well with half of the previously reported persistence length of HA ($L_{p,HA} = 4.1 \pm 0.4$ nm [2]). Moreover, the effective contour length shown in (A) is approximately half the contour length of the loop ($L_{c,eff} \approx L_{c,loop}/2$). Source data are provided as a Source Data file.

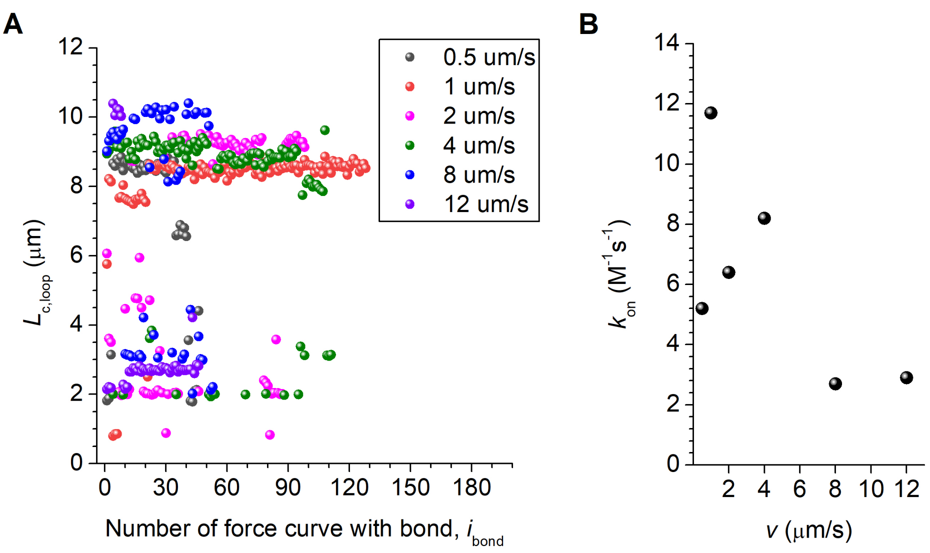

Supplementary Figure 8. Estimating k_{on} for LYVE-1 binding to HA loops. The DFS dataset linked to Figure 2A-C and Supplementary Figure 7 was used to estimate the rate of LYVE-1 binding to HA loops. To this end, the frequency of observing a bond was correlated with the local concentrations of binding sites on HA (defined by the size of the HA loop) and LYVE-1 on the surface, and the dwell time of the AFM tip at the surface (τ_{dwell}). (A) Evolution of the HA loop contour length, $L_{c,loop}$ (determined as described in Supplementary Figure 7) over the sequence of force curves for each of the approach/retract velocities tested. (B) Rate constant for bond formation k_{on} as a function of approach/retract velocity. Taken together, these data gave a weighted average (according to the total number of bonds per velocity) of $k_{on} = 5.5 \pm 3.0\ M^{-1}s^{-1}$.

To calculate k_{on}, we approximated the loop as a random coil with radius of gyration $R_g \propto L_{c,loop}^{3/5}$, and used the values $R_{g,ref} = 75$ nm and $L_{c,ref} = 2.2$ µm for an 840 kDa HA chain [2] as a reference, such that $R_g = R_{g,ref}(L_{c,loop}/L_{c,ref})^{3/5}$. We further approximate an HA octasaccharide as the footprint of LYVE-1 on the HA chain. With a contour length of 1 nm per disaccharide, the length of the footprint is $L_{c,fp} = 4$ nm, and the number of independent binding sites per HA loop is $N_{bs} = L_{c,loop}/L_{c,fp}$. The average number of bonds per force curve is defined by the concentration of binding sites in the HA loop, $c_{bs} \approx N_{bs}/(\frac{4}{3}\pi N_A R_g^3)$ (where N_A is Avogadro's number), the number of receptors within reach of the HA loop, $N_R \approx \pi R_g^2/d_{rms,R}^2$ (where $d_{rms,R}$ is the root-mean-square distance between receptors), the binding rate constant k_{on}, and the contact time, $\tau_{contact} \approx \tau_{dwell}$, as $n_{bonds/curve} = c_{bs} N_R k_{on} \tau_{contact} \approx \frac{3 N_{bs} k_{on} \tau_{dwell}}{4 R_g d_{rms,R}^2 N_A} = \frac{3 L_{c,loop}^{2/5} L_{c,ref}^{3/5} k_{on} \tau_{dwell}}{4 L_{c,fp} R_{g,ref} d_{rms,R}^2 N_A}$. Hence, $k_{on} \approx \frac{4 d_{rms,R}^2 N_A}{3 \tau_{dwell}} \frac{L_{c,fp} R_{g,ref} n_{bonds/curve}}{L_{c,ref}^{3/5} L_{c,loop}^{2/5}}$.

In our experiments, we found the size of the HA loop to vary to over time, presumably due to occasional changes in the bonds between biotin on HA-*g*-b and streptavidin on the AFM tip that define the loop size. However, these changes tended to be relatively rare (*i.e.*, apart from a few exceptions, many consecutive binding events with identical HA loop size were typically seen before a change in loop size was detected; see panel A), and hence are unlikely to perturb the rupture force statistics. Moreover, virtually all force curves showed either one or no rupture event. Considering these findings, the rate constant was estimated as $k_{on} \approx \frac{4 d_{rms,R}^2 N_A}{3 \tau_{dwell}} \frac{L_{c,fp} R_{g,ref}}{L_{c,ref}^{3/5} n_{total}} \sum_{i=1}^{n_{bond}} \frac{1}{L_{c,loop}^{2/5}(i)}$, where n_{total} is the total number of force curves acquired and n_{bond} the number of force curves with a rupture event.

For the present experiment, $d_{\mathrm{rms,R}} = d_{\mathrm{rms,ref}} \left(\frac{c_{\mathrm{ref}}}{c_{\mathrm{R}}}\right)^{1/2} \left(\frac{t_{\mathrm{ref}}}{t_{\mathrm{R}}}\right)^{1/4} = 28$ nm was estimated considering that LYVE-1 binding is mass-transport limited (*i.e.*, $d_{\mathrm{rms}}^{-2} \propto ct^{1/2}$ in stagnant solution, as used here [3]) with $c_{\mathrm{R}} = 1.0\ \mu\mathrm{g/mL}$ and $t_{\mathrm{R}} = 15$ min (see Supplementary Figure 7), and $d_{\mathrm{rms,ref}} = 50$ nm for $c_{\mathrm{ref}} = 0.25\ \mu\mathrm{g/mL}$ and $t_{\mathrm{ref}} = 25$ min estimated previously for a protein (CD44 ectodomain) of comparable size [2]. n_{total} was 180, 201, 344, 262, 403 and 430 for velocities of 0.5, 1, 2, 4, 8 and 12 µm/s, respectively, and $\tau_{\mathrm{dwell}} = 3$ s. All other parameters are defined in panel A and the present caption. Source data are provided as a Source Data file.

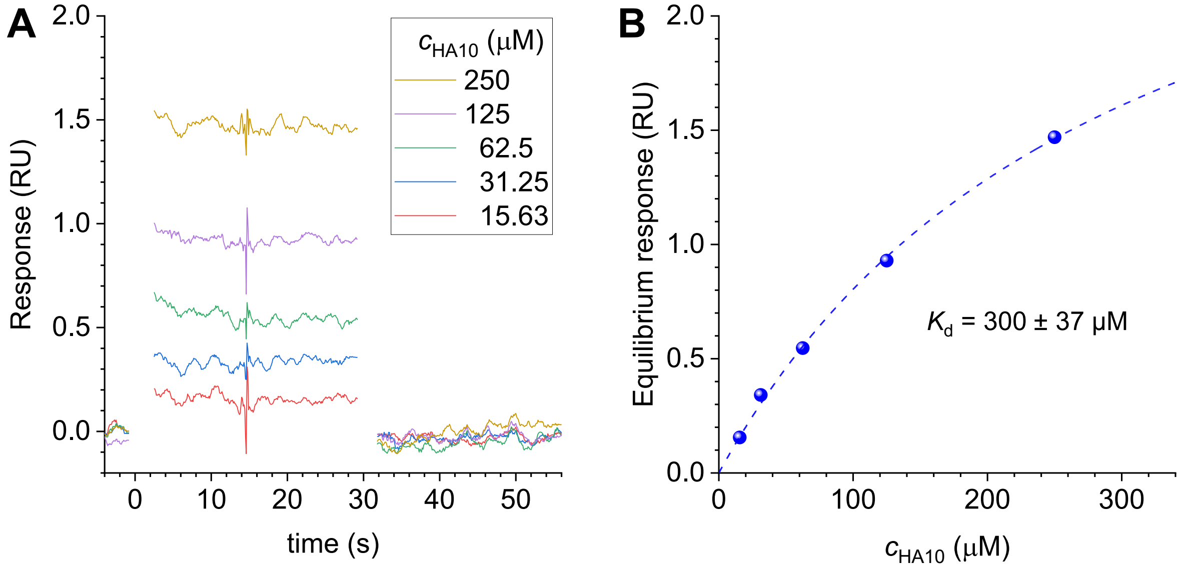

Supplementary Figure 9. LYVE-1•HA interaction affinity quantified by surface plasmon resonance (SPR). In this assay, immobilised LYVE-1 served as the ligand, and binding of HA decasaccharides (HA10) as soluble ligands was monitored with a Biacore 1K+ (Cytiva). (A) Representative SPR sensorgram showing dose dependent binding during the oHA incubation phase (0 to 30 s), and full elution during the rinsing phase (>30 s). Binding and unbinding processes were too rapid for binding rates to be quantified. Assay conditions: hLYVE-1 Δ238 with a biotin/His_{10} dual tag was immobilised *via* the biotin tag to an SPR chip presenting streptavidin in a methyl dextran matrix (Series S Sensor Chip SA; Cytiva # 29104992) at a response level of 2300 RU. A reference channel without immobilised LYVE-1 was used to correct for any background (*e.g.*, due to refractive index variations in the presence of oHA). (B) Equilibrium responses as a function of oHA concentration c_{oHA} for the data shown in panel A (sphere symbols) with a best-fit Langmuir isotherm (dashed line) to extract the indicated affinity, K_d. Analysis of 6 datasets with 2 different LYVE-1 surface densities (1000 RU and 2300 RU) gave K_d = 227 ± 76 µM. Source data are provided as a Source Data file.

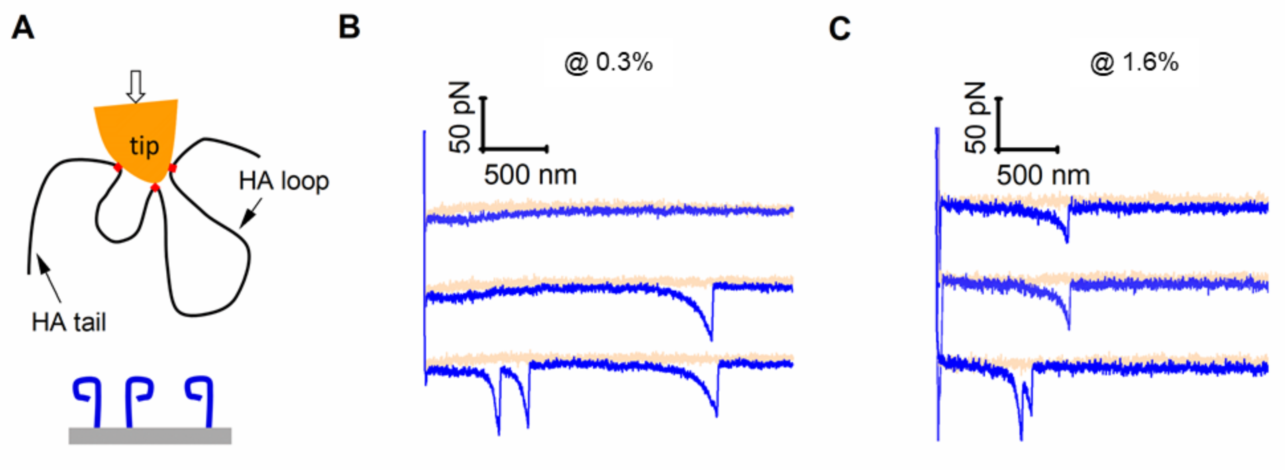

Supplementary Figure 10. LYVE-1 binds loops and tails in distinctly different ways which can co-exist on the same HA chain. (A) Schematic illustration of the DFS setup to probe interactions of HA loops with LYVE-1 at high surface density. The HA chains used in this assay were biotinylated at random positions along the chain (HA-*g*-b), and biotin-mediated anchorage to the AFM probe gives rise to one or several loops and two tails per chain. The average size of the loops (and tails) was controlled by the degree of biotinylation, indicated in panels B and C as nominal % values (see Methods for details). (B) Three representative force *vs.* distance curves (offset along the *y* axis for clarity; *pink* – tip approach, blue – tip retract) obtained at 0.3% HA biotinylation. The curves show the response characteristic of deterministic binding to HA tails as in Figure 1C, albeit at lower magnitude owing to a lower LYVE-1 surface density (*cf.* Supplementary Figure 4B; note the consistency in curve shape and in magnitude of forces and interaction range across the three curves); in addition a varying number of chain stretching and bond rupture events (0, 1 and 3, respectively, from top to bottom) characteristic for stochastic binding to loops (as in Figure 2B) can be discerned. (C) Three representative force *vs.* distance curves obtained at 1.6% HA biotinylation, with 1 (top two curves) or 2 (bottom curve) chain stretching and bond rupture events. The magnitude of the tail binding response is seen to disappear within the experimental noise, *i.e.*, as tails tend to be shorter and the LYVE-1 surface density lower. Assay conditions: HA-*g*-b incubation: 2 µg/mL for 6 min (all samples); hLYVE-1 Δ238 with a biotin/His_{10} dual tag incubated on His-tag-capturing PEG: 2 µg/mL for 30 min (for 0.3% HA-*g*-b samples; n = 213) and 0.7 µg/mL for 20 min (for 1.6%; n = 513); approach/retract velocity: 0.5 µm/s (for 0.3%) and 1 µm/s (for 1.6%); surface dwell time: 2 s (for 0.3%) and 3 s (for 1.6%). Data are representative of two independent experiments with distinct HA-coated probes and receptor-coated substrates per condition. Source data are provided as a Source Data file.

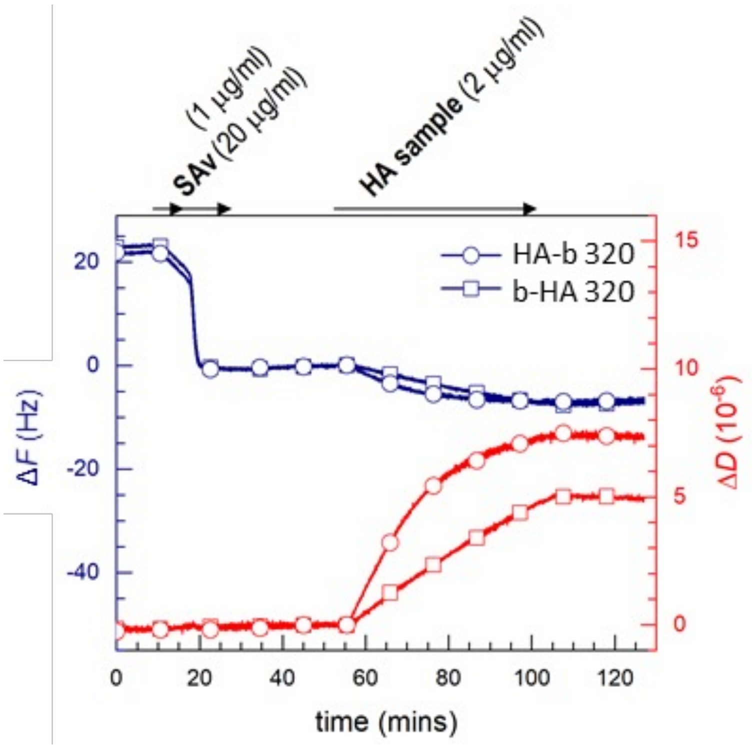

Supplementary Figure 11. QCM-D characterisation of HA anchorage *via* a biotin at the reducing (HA-b 320) and non-reducing (b-HA 320) end, respectively. QCM-D frequency shifts (ΔF) are shown as blue lines, and dissipation shifts (ΔD) as red lines with open squares. The start and duration of incubation with different samples are indicated by arrows on top of the graphs along with the incubation concentrations; remaining times represent washing steps with working buffer. Gold-coated QCM-D sensors were coated *ex situ* with a biotin-displaying OEG monolayer (as described for AFM probes in Methods), prior to the *in situ* deposition steps (as indicated). QCM-D responses demonstrate binding of both HA constructs. A reduced initial binding of b-HA 320 was consistently observed, by a factor 1.9 ± 0.1 (n = 2 independent experiments) compared to HA-b 320, which we attribute to a correspondingly reduced effective concentration of biotinylated HA. A similar ratio of HA surface densities was expected for the DFS assays (Figure 2D-G), as both HA constructs were incubated at 2 μg/mL for 6 min; forces displayed in Figure 2G were increased by a factor of 1.9 to account for the reduced HA coverage. Source data are provided as a Source Data file.

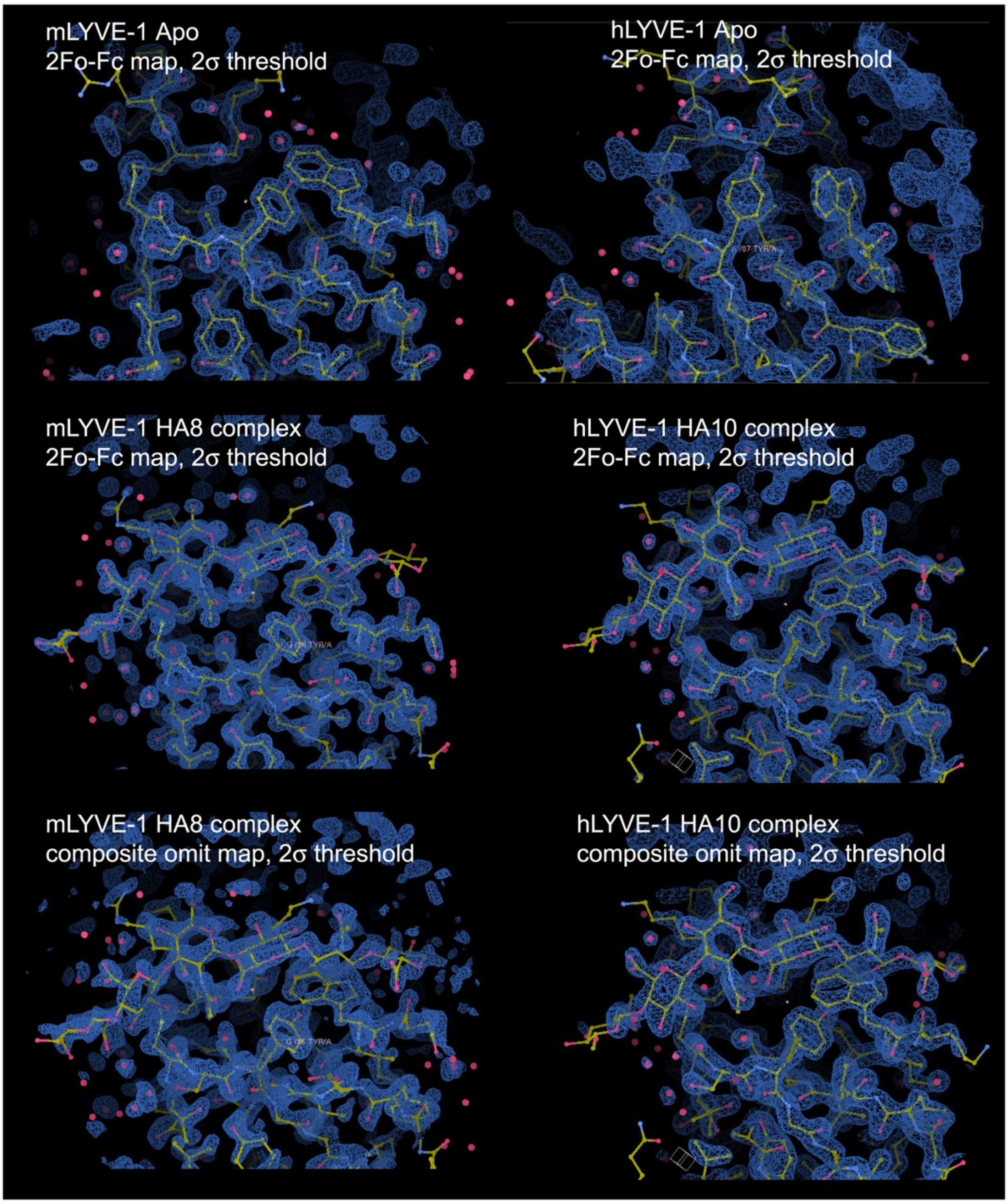

Supplementary Figure 12. X-ray density maps for LYVE-1 structures in Apo and complexed states. Gallery of density maps, all ar 2σ, for 2Fo-Fc maps and a composite omit map calculated for the HA complexes using Phenix software.

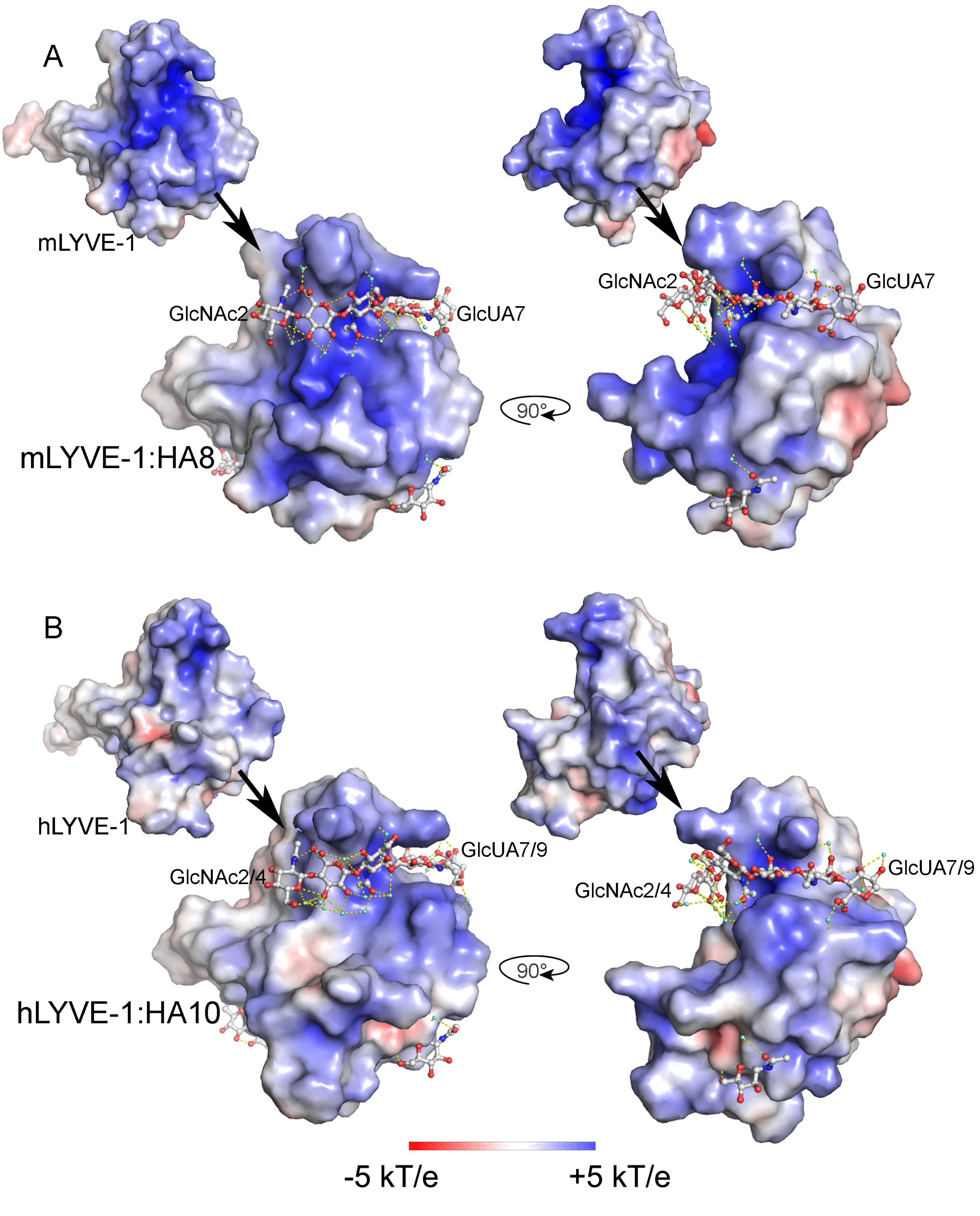

Supplementary Figure 13. Electrostatic surface representations. mLYVE-1 (A) and hLYVE-1 (B) with orthogonal views and showing for each panel the unbound (smaller) and HA-bound (larger) structures. Surfaces are coloured -5 $k_B T/e$ (red) to +5 $k_B T/e$ (blue) (as shown in the key) and were calculated using APBS and rendered in Pymol (The PyMOL Molecular Graphics System, Version 2.0 Schrödinger, LLC).

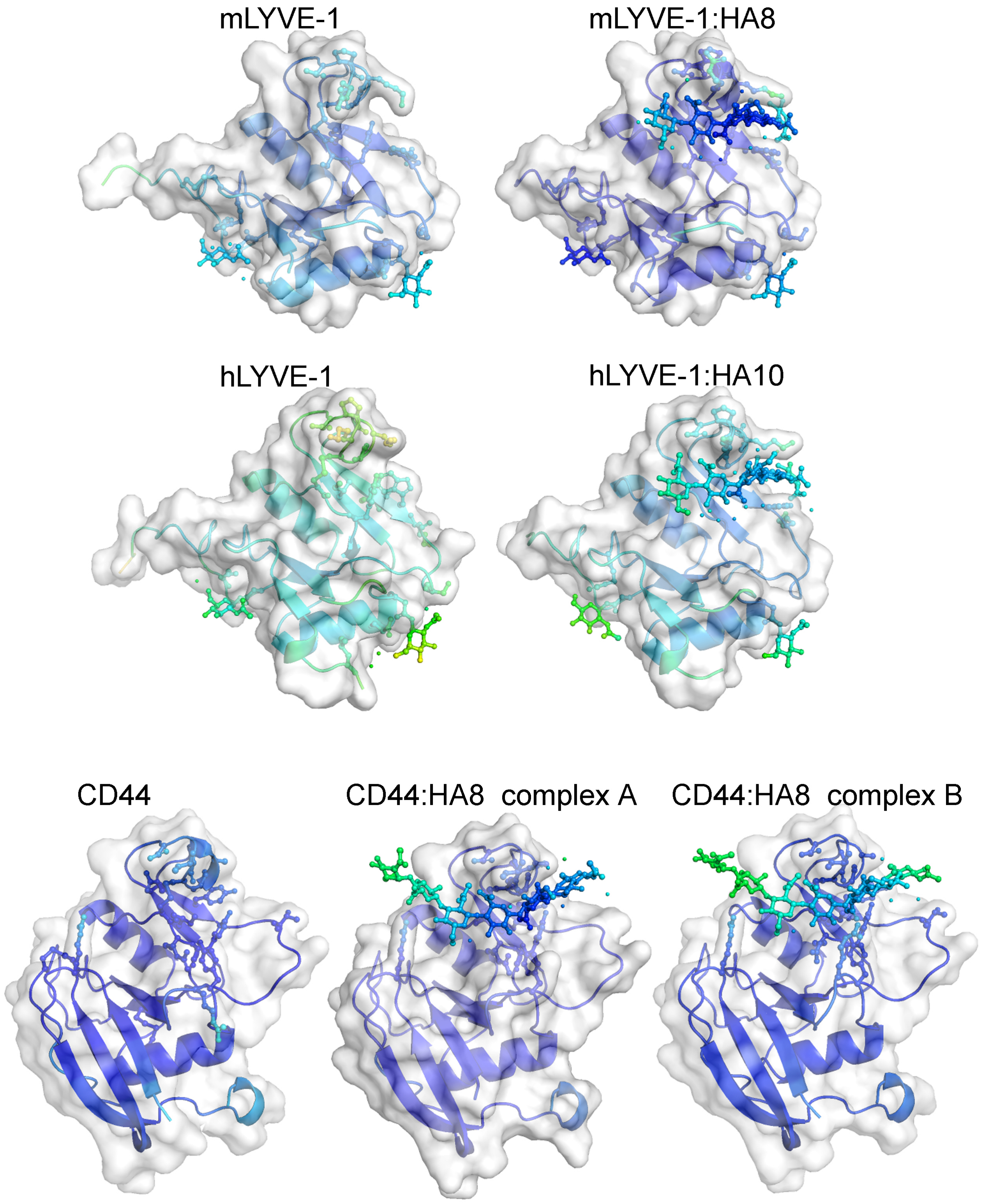

Supplementary Figure 14. B factor variation representations. Depictions of the crystallographic B factors of unbound and HA-bound forms of mLYVE-1, hLYVE-1 and mCD44 (complex A, 2JCQ, [https://doi.org/10.2210/pdb2JCQ/pdb]; complex B, 2JCR, [https://doi.org/10.2210/pdb2JCR/pdb]). Water molecules depicted in other figures are included here also. All structures are on the same scale; B factors vary from 2.0 Å^2 (blue) to 119.2 Å^2 (orange) in reverse rainbow order.

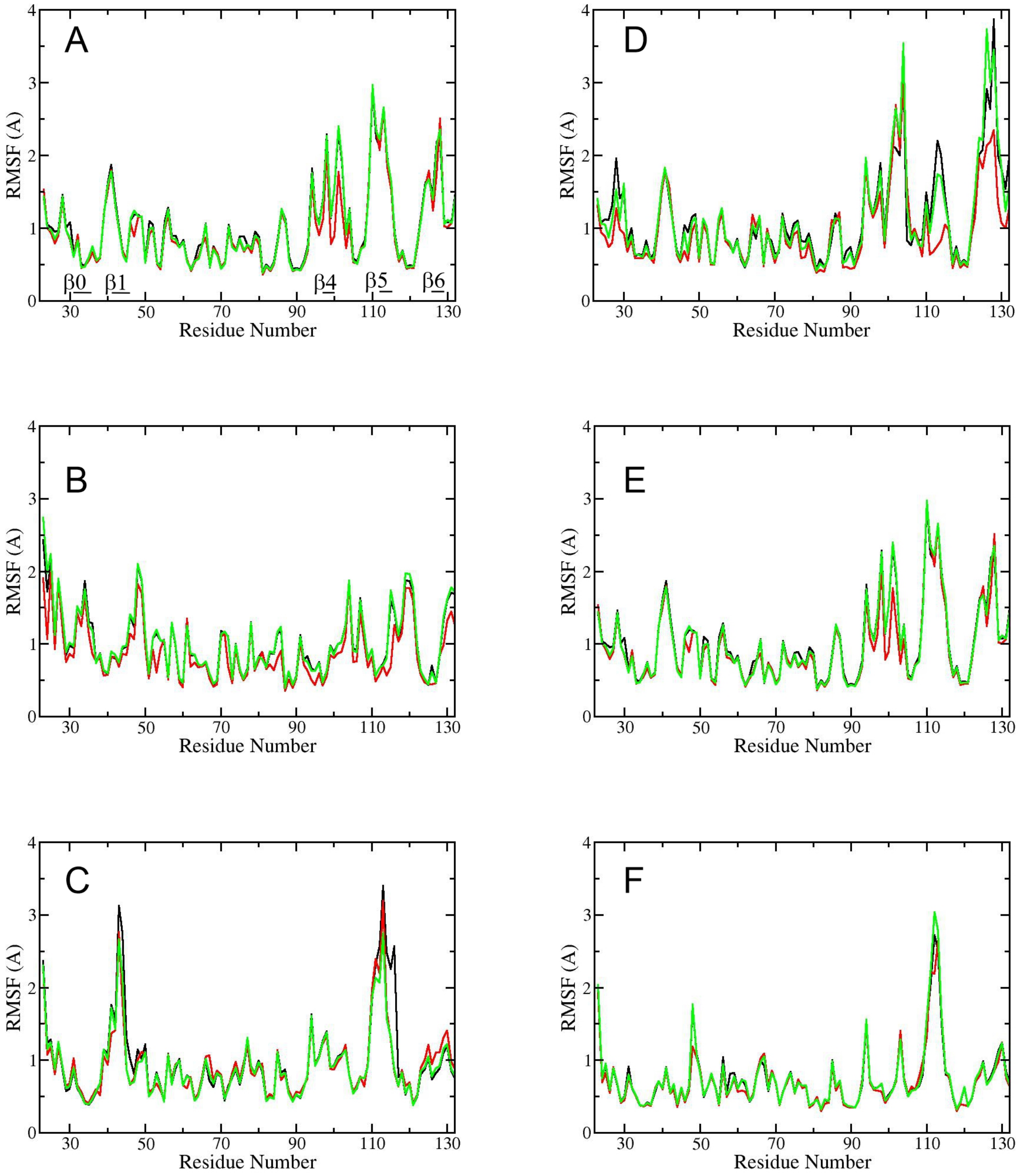

Supplementary Figure 15. The effect of HA binding on receptor amino acid positional fluctuations. Shown are root-mean-square fluctuations (RMSF) in the unliganded apoprotein (panels A-C) and HA-bound receptor complexes (panels D-F) for mLYVE-1 (A, D), hLYVE-1 (B, E) and mCD44 (C, F) as derived from molecular dynamics (MD) simulations. To show that the RMSF values are equilibrated, we present data from 100 ns (red), 300 ns (green) and 500 ns (black) portions of MD trajectories. Source data are provided as a Source Data file.

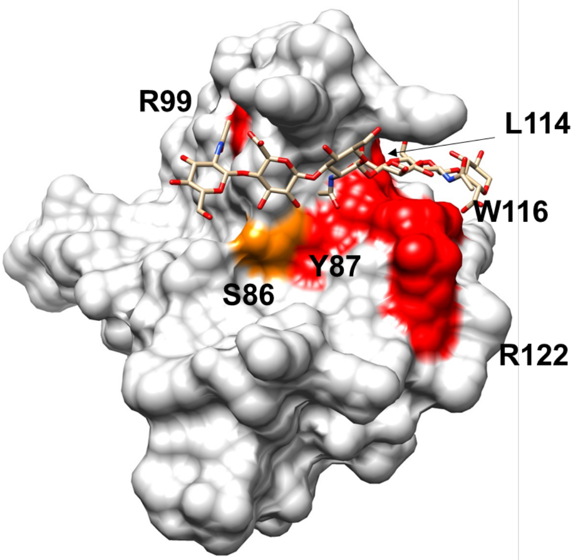

Supplementary Figure 16. Binding epitopes of the anti-hLYVE-1 function-blocking monoclonal antibody 3A. Binding epitopes (red) defined by prior mutagenesis studies [4] are mapped onto the crystal structure of the hLYVE-1 Δ144 HA10 complex (red – high contribution to binding, orange – low contribution to binding).

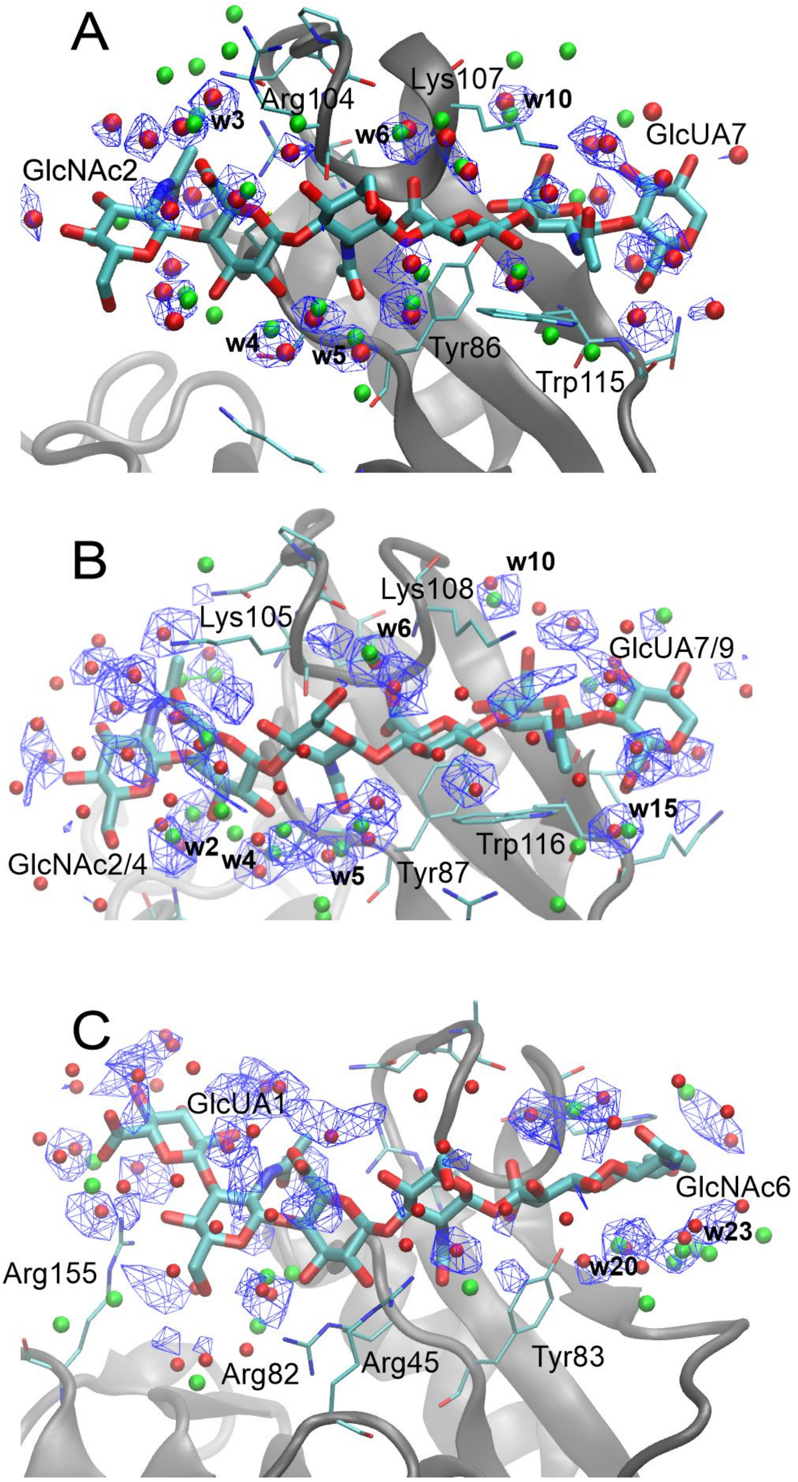

Supplementary Figure 17. Comparison of water positions obtained by MD simulations *vs.* crystallography. Calculated water positions (*red spheres*) and densities (*blue mesh*) from 10ns restrained MD (see Methods) are shown for mLYVE-1 (A), hLYVE-1 (B) and CD44 (C), compared with the crystallographic water positions (*green spheres*). Sugar residues of the bound HA ligand are shown as thick sticks and amino acid residues critical for HA binding are shown as thin sticks (coloured *turquoise* in each case). Water molecule numbering follows that from Figure 5, and Supplementary Table 2. Figure rendered with VMD, version 1.9.3 [5].

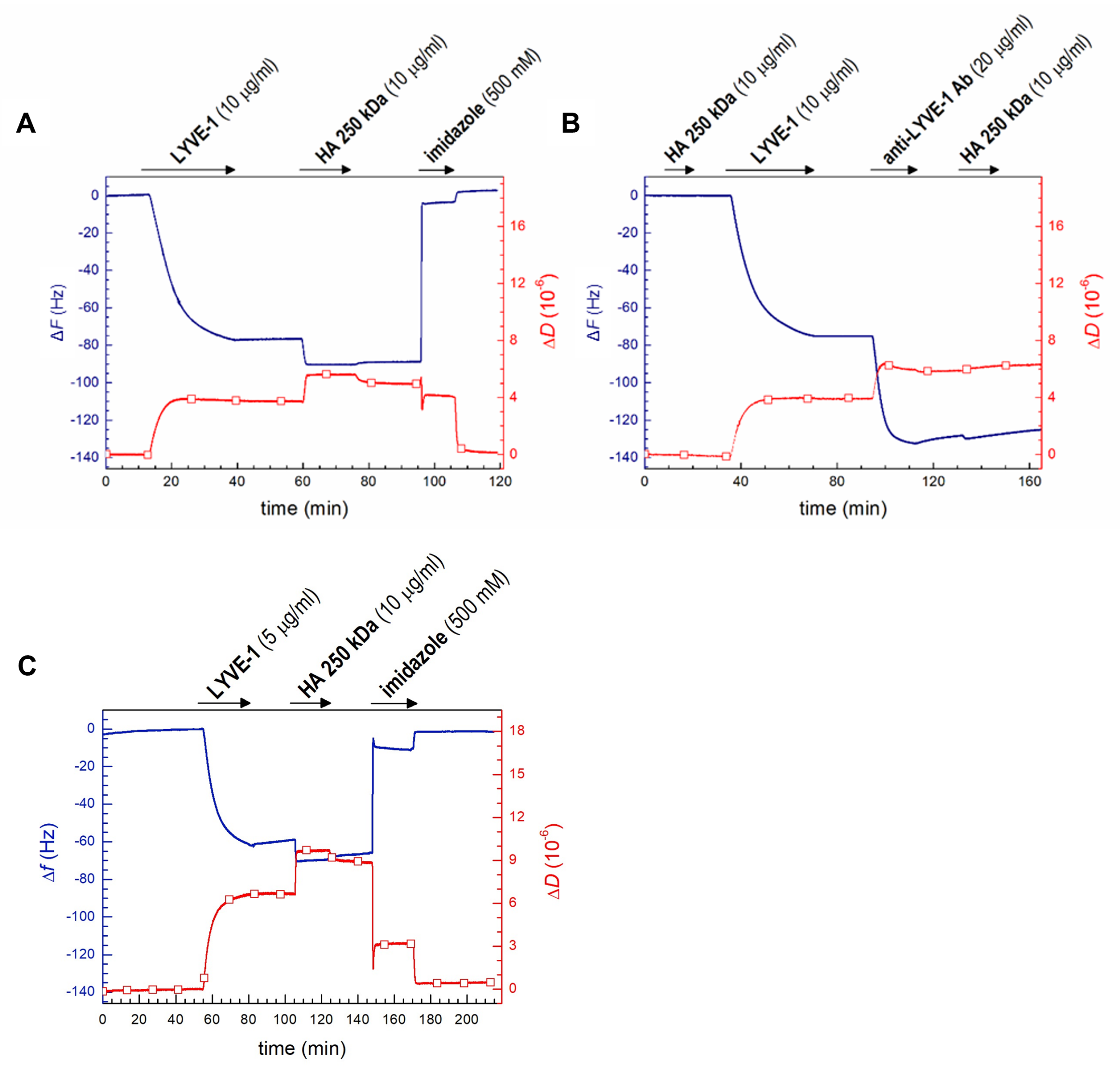

Supplementary Figure 18. QCM-D characterisation of LYVE-1 immobilisation on His-tag-capturing PEG substrates. QCM-D frequency shifts (ΔF) are shown as *blue lines*, and dissipation shifts (ΔD) as *red lines with open squares*. The start and duration of incubation with different samples are indicated by arrows on top of the graphs along with the incubation concentrations; remaining times represent washing steps with working buffer. QCM-D responses in (A) and (C) indicate formation of a stable LYVE-1 monolayer; they also demonstrate that LYVE-1 is specifically immobilised through its His tag (*i.e.,* it can be fully eluted in imidazole) and that it successfully binds soluble HA (here 250 kDa). Changes in ΔF and ΔD at the end of the imidazole incubation (*i.e.*, at 107 min in (A) and 170 min in (C) do not reflect any changes on the surface but result from changes in the viscosity and/or density of the surrounding solution owing to the prior presence of imidazole. Data in (B) demonstrate that HA binds exclusively through the authentic HA-binding site on LYVE-1, *i.e.*, HA does not bind to the His-tag capturing substrate without LYVE-1, and HA binding to LYVE-1 can be blocked with anti-LYVE-1 Ab. Conditions: (A-B) hLYVE Δ238 with a His_{10} single tag; (C) hLYVE-1 Δ238 with a biotin/His_{10} dual tag. Data are representative of two independent experiments with distinct substrates per condition. Source data are provided as a Source Data file.

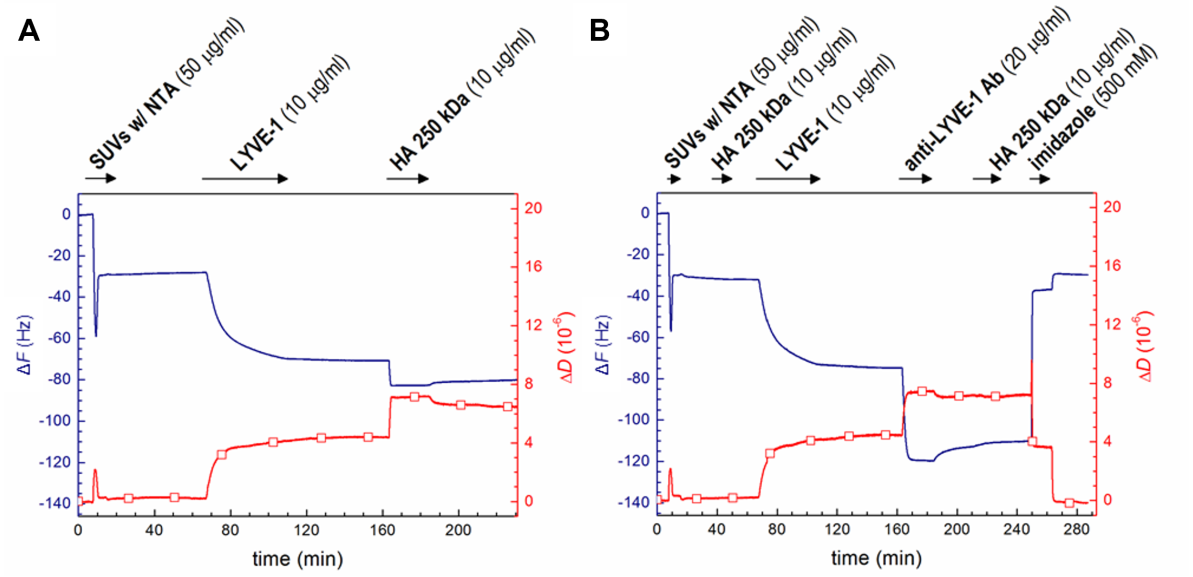

Supplementary Figure 19. QCM-D characterisation of LYVE-1 anchorage on His-tag-capturing supported lipid bilayers (SLBs). Data are presented analogous to Supplementary Figure 18. SLBs were formed by spreading of small unilamellar vesicles (SUVs) composed of a DOPC:(NTA)$_3$-SOA mixture (molar ratio 19:1) on silica-coated substrates; the two-phase responses in ΔF and ΔD, the small final dissipation shift ($< 0.3 \times 10^{-6}$) and the final frequency shift (-29 ± 1 Hz) are characteristic for the formation of SLBs of good quality [2,6]. QCM-D responses in (A) furthermore indicate formation of a stable, HA-binding LYVE-1 monolayer, and demonstrate that LYVE-1 is specifically immobilised through its His tag (*i.e.*, it can be fully eluted in imidazole). Data in (B) demonstrate that HA binds exclusively through the authentic HA-binding site on LYVE-1, *i.e.*, HA does not bind to LYVE-1-free SLBs and HA binding to LYVE-1 is blocked with anti-LYVE-1 Ab. Conditions: hLYVE Δ238 with a His$_{10}$ single tag was used throughout. Data are representative of two independent experiments with distinct substrates per condition. Source data are provided as a Source Data file.

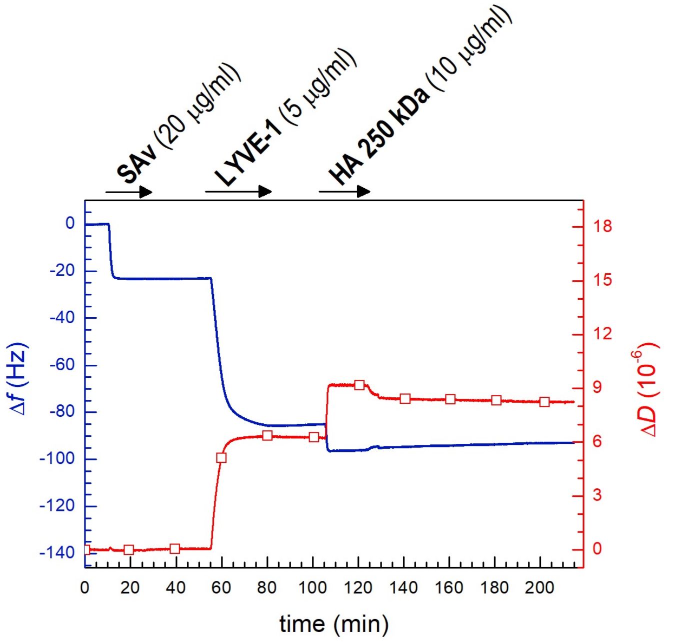

Supplementary Figure 20. QCM-D characterisation of LYVE-1 anchorage on a streptavidin monolayer. Data are presented analogous to Supplementary Figure 18. A streptavidin (SAv) monolayer was formed on a biotin-displaying OEG monolayer as already shown in Supplementary Figure 11. Further QCM-D responses demonstrate that hLYVE-1 Δ238 with a biotin/His_{10} dual tag is stably immobilised through its biotin tag and can actively bind HA. The LYVE-1 and HA binding responses are comparable for immobilisation *via* the biotin (shown here) or His_{10} tag (shown in Supplementary Figure 18C), demonstrating comparable protein presentation and HA binding activity in these two assays. Data are representative of two independent experiments with distinct substrates per condition. Source data are provided as a Source Data file.

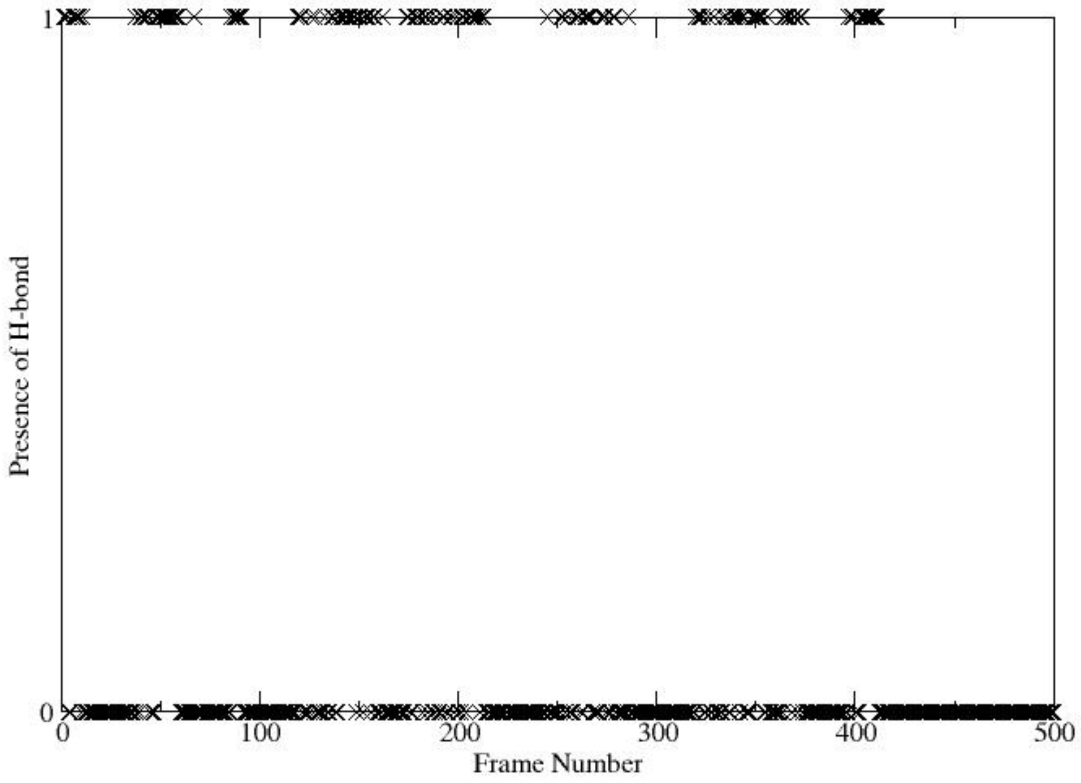

Supplementary Figure 21. Timescale of H-bonds studied by MD. The presence/absence of a selected H-bond (Gly106:N…GlcUA5:O6B) in the mLYVE-1/HA6 complex throughout 500 ns MD replica 2 showing that regular sampling sufficiently populates both states. Source data are provided as a Source Data file.

Supplementary Tables

Supplementary Table 1. Crystallographic data and refinement statistics. Values in parentheses are for the highest resolution shell.

	mLYVE-1 Apo PDB ID 8ORX	hLYVE-1 Apo PDB ID 8OS2	mLYVE-1 + HA8 PDB ID 8OX3	hLYVE1 + HA10 PDB ID 8OXD
**Data collection statistics**				
Wavelength (Å)	0.9686	0.9686	0.9686	0.9159
Resolution range (Å)	29.16 - 1.54 (1.64 - 1.54)	41.77 - 1.64 (1.71 - 1.64)	46.9 - 1.05 (1.07 - 1.05)	37.83 - 1.32 (1.37 - 1.32)
Space group	C 1 2 1	P 41 21 2	C 1 2 1	P 21 21 21
Unit cell dimensions(Å) (°)	31.37 58.32 52.35 90 94.62 90	70.81 70.81 75.77 90 90 90	86.11 56.96 30.91 90 106.27 90	39.59 53.26 53.74 90 90 90
Total reflections	90831	303695	293894	184719
Unique reflections	13957 (2315)	24215 (2943)	46051 (284)	27198 (2673)
Multiplicity	6.5	12.5	6.4	10.4
Completeness (%)	99.90 (99.78)	99.97 (100.00)	69.05 (6.88)*	99.35 (98.85)
Mean I/sigma(I)	16	21	11.1	15.1
Wilson B-factor	16.73	30.02	7.9	16.19
R-meas	0.067	0.059	0.101	0.084
CC1/2	0.999 (0.954)	1.000 (0.498)	0.995 (0.378)	0.999 (0.683)
**Structure refinement statistics**				
Reflections used in refinement	13957 (2315)	24215 (2943)	46051 (284)	27198 (2673)
Reflections used for R-free	775 (152)	1180 (147)	2211 (9)	1384 (137)
R-work	0.1692 (0.2063)	0.1850 (0.3742)	0.1499 (0.3045)	0.1730 (0.3326)
R-free	0.1947 (0.2473)	0.2227 (0.3622)	0.1621 (0.3936)	0.2178 (0.3245)
Number of non-hydrogen atoms	1045	1160	1221	1125
macromolecules	883	962	908	914
ligands	28	40	140	120
solvent	134	158	173	91
Protein residues	115	123	113	119
RMS(bonds)	0.006	0.007	0.012	0.012
RMS(angles)	0.86	1.03	1.19	1.29
Ramachandran favored (%)	97.35	98.35	96.4	97.44
Ramachandran allowed (%)	2.65	1.65	3.6	2.56
Ramachandran outliers (%)	0	0	0	0
Rotamer outliers (%)	0	0.93	0.97	0.98
Clashscore	0.56	4.48	3.41	9.78
Average B-factor	20.01	39.97	14.03	25.92
macromolecules	18.55	37.67	10.44	23.41
ligands	23.94	58.58	20.95	34.67
solvent	28.82	49.3	27.26	39.6

*The low completeness of the outer shell for this dataset relates to the narrowness of the shell. One fewer shell results in an outer segment of 1.13-1.05Å which is 14.68% complete.

Supplementary Table 2. Direct and indirect interactions between HA and LYVE-1 or CD44 as observed in crystallographic structures. Waters are only listed if they are bound by more than one atom of a ligand (be it the protein or HA) and therefore contribute to the structure of the bound HA. > and < indicate hydrogen bond donation where it is possible to define it; -- indicates hydrogen bonds where the polarity cannot be ascertained. Direct bonding interactions conserved between the murine and human complexes are marked with *. Water residues (Wat) in bold are conserved between mLYVE-1 and hLYVE-1 HA structures and, in one case (w13), with the CD44-HA structure also. Four of these (w4, w5, w6 and w10) are conserved not only in position but in forming the equivalent interactions in each case. Water molecule numbers in the deposited PDB files for LYVE-1 structures match the water numbers for each structure in this table. Where interacting atoms are given, the HA contributors are coloured red.

mLYVE1-HA	Direct H-bonds	Water-mediated H-bonds	HA-only waters bound	Hydrophobic interactions
GlcNAc2			O7>Wat1--O3<br>O5--**Wat2**	Asn102/Cys105 *
GlcUA3		Asn102 (O6B<Wat3(>OD1)(<ND2))<br>Arg104 (O6B<Wat3<NE)<br>Ser85 ((O2)(O3)--**Wat4**--OG)[a]<br>Cys84 ((O2)(O3)--**Wat4**>O)	O3--**Wat2**--O4	
GlcNAc4	Arg104 (N2>O)*,[c]	Tyr86 (O7<**Wat5**--N)[b]	O7<**Wat7**--O5--Wat8	Cys84/Cys105 *
GlcUA5	Gly106 (O6B<N) *,[d]<br>Lys107 (O6A<N) *	Pro103 (O6A--**Wat6**>O)	Wat8--(O3)(O4), O4--**Wat7**<br>Wat9--(O2)(O3)	
GlcNAc6	Lys107 (O3--NZ + O7<NZ)<br>Tyr86 (O4--OH) *	Gly106 (O6--**Wat10**>O)		Ile96/Trp115 *
GlcUA7	Asn116 (O6A<N) *			
**hLYVE1-HA**	**Direct H-bonds**	**Water-mediated H-bonds**	**HA-only waters bound**	**Hydrophobic interactions**
GlcNAc2/4		Cys85 (O5--**Wat2**>O)	O6--Wat11	Asn103/Cys106 *
GlcUA3/5		Cys85 ((O2)(O3)--**Wat4**>O[a] +<br>O3--Wat12>O + O3--**Wat2**>O)	O3--Wat11	
GlcNAc4/6	Lys105(N2>O) *,[c]	Tyr87 (O7<**Wat5**--N)[b]	**Wat13**--(O3)(O4), O3--Wat14<br>**Wat7**>O7	Ile97/Cys85/Cys106 *
GlcUA5/7	Gly107 (O6B<N) *,[d]<br>Lys108 (O6A<N) *	Pro104 (O6A--**Wat6**>O)		
GlcNAc6/8	Tyr87 (O4--OH)*	Gly107 (O6--**Wat10**>O)		Ile97/Trp116 *
GlcUA7/9	Lys117 (O6A<N) *	Ile115 (O3--Wat16>O)<br>Lys117 (O6B<Wat15>O)	O2--Wat17--O3	

CD44 crystal A (2JCQ)	Direct H-bonds	Water-mediated H-bonds	HA-only waters bound	Hydrophobic interactions
GlcNAc2				
GlcUA3				
GlcNAc4			O3--**Wat2**--O5	Asn98/Cys101 *
GlcUA5[g]	Arg82 (O2--NH1)[a]		O3--**Wat2**--O4<br>O5--**Wat13**>O6A	
GlcNAc6	Tyr46 (O7<OH)[b]<br>Ile100 (N2>O)[c]		O4--**Wat13**	Ile92/Cys81/Cys101 *
GlcUA7	Tyr83 (O6B<OH)<br>Ala103 (O6A<N)[d]		Wat18--(O5)(O6A)	
GlcNAc8	Tyr109 (O6--OH)	Tyr83 (O4--Wat20--OH)	O3--Wat19<br>N2--Wat21>O7	
**CD44 crystal B (2JCR)**	**Direct H-bonds**	**Water-mediated H-bonds**	**HA-only waters bound**	**Hydrophobic interactions[f]**
GlcNAc2				
GlcUA3				
GlcNAc4			O5--**Wat2**--O6	Asn98/Cys101 *
GlcUA5	Arg82 (O2--NE[a] and O3--NE)<br>Cys81 (O2>O)<br>Arg45 (O2--(NE)(NH2))		O3--**Wat2**	
GlcNAc6	Arg45 (O3<NH2 and O7<NH2)<br>Tyr46 (O7<OH)[b]<br>Ile100 (N2>O)[c]			Ile92/Cys81/Cys101 *
GlcUA7	Tyr83 (O6B<OH)<br>Ala102 (O6A<N)[d]<br>Ala103 (O6A<N)		Wat22--(O5)(O6A) [e]	
GlcNAc8	Tyr109 (O6--OH)	Tyr83 (O4--Wat20--OH)<br>Thr113 (N2--Wat23>O)	O3--Wat22	

[a] Equivalent interactions of mLYVE Ser85 water bridge through Wat4 and hLYVE Cys85 water bridge through Wat4, to the direct hydrogen bond involving Arg82 in CD44.
[b] Equivalent interactions of mTyr86/hTyr87 main chain amino group to O7 of GlcNAc4/6 and direct hydrogen bonding of CD44 Tyr46 to GlcNAc6.
[c] The carbonyl oxygen-mediated bond to GlcNAc4/6 is equivalent with mLYVE-1 Arg105, hLYVE-1 Lys105 and CD44 Ile100.
[d] Bonding of the amino groups of mLYVE-1 Gly106, hLYVE-1 Gly106 and CD44 Ala102 are equivalent (though the sugar is apparently twisted differently between LYVE-1 and CD44 structures).
[e] Replaces Wat18 and Wat19 in 2JCQ.
[f] Hydrophobic areas of interaction estimated at 116 Å^2 in both mouse and human LYVE-1, and 59 Å^2 in mouse CD44 (2JCR).
[g] By super-position GlcUA5 in the CD44 structures aligns with GlcUA3/5 in the LYVE-1 structures.

Supplementary Table 3. Root-mean-square fluctuations, RMSF (Å) of the key HA contact residues of mLYVE/hLYVE (A), mCD44 (B) and of HA residues (C) as obtained from MD simulations.

A.

	mTyr86/ hTyr87 (β3)	mArg104/ hLys105 (β4/5 loop)	mLys107/ hLys108 (β4/5 loop)	mTrp115/ hTrp116 (β5/6 loop)	**average ± SD**
apoprotein	0.88/1.07	2.28/1.76	1.57/1.66	1.29/1.62	
complex with HA	0.68/0.61	1.89/1.29	2.13/1.66	0.85/0.64	
**apo/complex ratio**	**1.30/1.76**	**1.20/1.37**	**0.74/1.01**	**1.51/2.54**	**1.19 ± 0.33/ 1.67 ± 0.66**

B.

	Arg45	Tyr46	Arg82	Tyr83	Tyr109	**average ± SD**
apoprotein	1.81	1.36	1.04	0.83	0.75	
complex with HA	0.58	0.40	0.41	0.45	0.59	
**apo/complex ratio**	**3.11**	**3.45**	**2.56**	**1.83**	**1.28**	**2.45 ± 0.90**

C.

	mLYVE	hLYVE	mCD44
GlcUA1	n.d.	n.d.	3.65
GlcNAc2	2.58	3.09	1.84
GlcUA3	1.66	1.95	0.71
GlcNAc4	1.45	1.60	0.62
GlcUA5	1.43	1.48	0.78
GlcNAc6	1.36	1.60	1.58
GlcUA7	1.96	2.43	n.d.
**Average**	**1.74 ± 0.47**	**2.03 ± 0.63**	**1.53 ± 1.16**

Supplementary Table 4. Occupancy throughout MD simulations of direct (A) and water-mediated (B) hydrogen bonding between HA and mLYVE-1, hLYVE-1 or CD44. Bonding patterns conserved across proteins are shown in the same row. Nomenclature as in Extended Data Table 2. Averages and standard deviations from three independent MD trajectories are stated where applicable. Standard deviations are smaller than 10% of the averages in most cases, indicating that the results are relatively independent of the initial configuration.

A.

HA monosaccharide	mLYVE·HA		hLYVE·HA		CD44·HA (2JCR)	
	H-bond	Occupancy (%)	H-bond	Occupancy (%)	H-bond	Occupancy (%)
GlcUA3					Arg45 (O2-- (NE)	78±22
					Arg45 (O2-- (NH2)	55±9
					Cys81 (O2>O)	100±0
					Arg82 (O2--NE)	8±2 [c]
					Arg82 (O3--NE)	99±1
GlcNAc4	Arg104 (N2>O)	96±2	Lys105(N2>O)	77±18 [a]	Arg45 (O3<NH2)	36±11
					Arg45 (O7<NH2)	49±19
					Tyr46 (O7<OH)	100±0
					Ile100 (N2>O)	99±0
					Tyr83 (O6A/B<OH)	79±35
	Gly106 (O6B<N)	62±5 [a]	Gly107 (O6B<N)	24±2 [a]	Ala102 (O6A/B<N)	110±53 [f]
GlcUA5	Lys107 (O6A<N)	70±3 [a]	Lys108 (O6A<N)	32±10 [a]	Ala103 (O6A/B<N)	76±38
	Tyr86 (O4--OH)	97±3	Tyr87 (O4--OH)	82±18 [a]		
GlcNAc6	Lys107 (O3--NZ)	8±6 [a, b, c]				
	Lys107 (O7<NZ)	0 [b,c]				
					Tyr109 (O6<OH)(O6>OH)	13±9
	Asn116 (O6A<N)	46 [d]	Lys117 (O6A<N)	4[c, d, e]		
GlcUA7						

B.

HA monosaccharide	mLYVE·HA		hLYVE·HA		CD44·HA (2JCR)	
	H-bond	Occupancy (%)	H-bond	Occupancy (%)	H-bond	Occupancy (%)
GlcNAc2			Cys85 (O5--**Wat2**>O)	19±12[a]		
GlcUA3	Asn102 (O6B<Wat3(>OD1)(<ND2))	19±1	Cys85 ((O2)(O3)--**Wat4**>O + O3--Wat12>O + O3--**Wat2**>O)	0[c]		
	Arg104 (O6B<Wat3<NE)	70±1				

	Ser85 ((O2)(O3)--**Wat4**--OG)	44±8				
	Cys84 ((O2)(O3)--**Wat4**>O)	18±9[a]1				
GlcNAc4	Tyr86 (O7<**Wat5**--N)	51±8	Tyr87 (O7<**Wat5**--N)	39±10[a]		
GlcUA5	Pro103 (O6A--**Wat6**>O)	83±4	Pro104 (O6A--**Wat6**>O)	71±14[a]		
GlcNAc6	Gly106 (O6--**Wat10**>O)	0[b,c]	Gly107 (O6--**Wat10**>O)	0[c]	Tyr83 (O4--Wat20--OH)	45±25
					Thr113 (N2--Wat23>O)	11[d]
GlcUA7			Ile115 (O3--Wat16>O)	12[d]		
			Lys117 (O6B<Wat15>O)	79±15[a]		

[a] H-bond present only in two of the three trajectories. The statistics takes into account only these two trajectories

[b] We note that MD shows over 100% occupancy of a non-crystallographic Lys107:NZ…GlcUA5:O6A/B salt bridge, likely due to erroneous overestimation of charged interactions (see the main text). This distorts the crystallographic HA binding mode and prevents the formation of some native H-bonds, both direct and water-mediated.

[c] Occupancy in MD below 10 % is considered as failed to reproduce the X-ray crystallographic observation.

[d] H-bond present only in one of the three trajectories. Statistics cannot be carried out

[e] We note that in place of this H-bond found crystallographically, MD shows 67% occupancy of a non-crystallographic Trp116:NE1…GlcUA7:O6A/B H-bond

[f] Occupancy in MD over 100 % is due to bifurcated H-bonding to GlcUA carboxylate

Supplementary References

1. Madeira, F., Pearce, M., Tivey, A.R.N., Basutkar, P., Lee, J., Edbali, O., Madhusoodanan, N., Kolesnikov, A. and Lopez, R. (2022). Search and sequence analysis tools services from EMBL-EBI in 2022. Nucleic Acids Res *50*, W276-W279. 10.1093/nar/gkac240.
2. Bano, F., Banerji, S., Howarth, M., Jackson, D. and Richter, R. (2016). A Single molecule assay to probe monovalent and multivalent bonds between hyaluronan and its key leukocyte receptor CD44 under force. Sci Rep *6*, 34176. 10.1038/srep34176
3. Kirichuk, O., Srimasorn, Zhang, S.X., Roberts, A.R.E., Coche-Guerente, L., Kwok, J.C.F., Bureau, L., Debarre, D. and Richter, R.P. Competitive Specific Anchorage of Molecules onto Surfaces: Quantitative Control of Grafting Densities and Contamination by Free Anchors (2023) Langmuir *39*, 18410-18423. 10.1021/acs.langmuir.3c02567
4. Banerji, S., Hide, B.R., James, J.R., Noble, M.E. and Jackson, D.G. (2010). Distinctive properties of the hyaluronan-binding domain in the lymphatic endothelial receptor Lyve-1 and their implications for receptor function. J Biol Chem *285*, 10724-10735. 10.1074/jbc.M109.047647.
5. Humphrey, W., Dalke, A. and Schulten, K. (1996). VMD: visual molecular dynamics. J Mol Graph *14*, 33-38, 27-38. 10.1016/0263-7855(96)00018-5.
6. Richter, R.P., Berat, R. and Brisson, A.R. (2006) Formation of solid-supported lipid bilayers: an integrated view. Langmuir *22*, 3497-3505. 10.1021/la052687c
