# Supplementary material for: Structure and unusual binding mechanism of the hyaluronan receptor LYVE-1 mediating leucocyte entry to lymphatics
Source: Nat Commun. 2025 Mar 20;16:2754. doi: 10.1038/s41467-025-57866-8 (PMC11926218; doi:10.1038/s41467-025-57866-8)
Supplement: Supplementary file 2 — Description of Additional Supplementary Files [file 41467_2025_57866_MOESM2_ESM.pdf]

**Description of Additional Supplementary Files**

**Supplementary Movie 1**. Morphing between the murine LYVE-1 apo structure (PDB ID 8ORX [https://doi.org/10.2210/pdb8ORX/pdb*])* and the HA8-bound form (PDB ID 8OX3 [https://doi.org/10.2210/pdb8ox3/pdb]), showing a semi-transparent molecular surface for protein and HA, with the mLYVE-1 backbone represented as ribbons, coloured rainbow-wise from blue at the amino-terminus to red at the carboxy-terminus, save for β0 (wheat coloured). Sidechains of critical residues, labelled in the first and last frames, move to allow end-on HA binding, as described in Main Text and Figures 3 and 4.

**Supplementary Movie 2**. Morphing between the human LYVE-1 apo structure(PDB ID 8OS2 [https://doi.org/10.2210/pdb8OS2/pdb]) and the HA10-bound form (PDB ID 8OXD [https://doi.org/10.2210/pdb8OXD/pdb]), showing a semi-transparent molecular surface for protein and HA, with the hLYVE-1 backbone represented as ribbons, coloured rainbow-wise from blue at the amino-terminus to red at the carboxy-terminus, save for β0 (wheat coloured). Sidechains of critical residues, labelled in the first and last frames, move to allow end-on HA binding, as described in Main Text and Figures 3 and 4.

**Supplementary Movie 3**. Morphing between the murine CD44 apo structure (PDB ID 2JCP [https://doi.org/10.2210/pdb2jcp/pdb]) and the higher resolution HA8 bound structure of CD44 (PDB ID 2JCQ [https://doi.org/10.2210/pdb2JCQ/pdb]), showing a semi-transparent molecular surface for protein and HA, with the mCD44 backbone represented as ribbons, coloured rainbow-wise from blue at the amino-terminus to red at the carboxy-terminus, save for β0 (wheat coloured) and β7 and β8 (pink). Sidechains of critical residues, labelled in the first and last frames, move after side-on HA binding, as described in Main Text.
