# Supplementary material for: Structure and unusual binding mechanism of the hyaluronan receptor LYVE-1 mediating leucocyte entry to lymphatics
Source: Nat Commun. 2025 Mar 20;16:2754. doi: 10.1038/s41467-025-57866-8 (PMC11926218; doi:10.1038/s41467-025-57866-8)
Supplement: Supplementary file 6 — Reporting Summary [file 41467_2025_57866_MOESM6_ESM.pdf]

nature portfolio

Corresponding author(s): David G.Jackson, Ralf R. Richter, Martin Lepsik, Robert Gilbert

Last updated by author(s): 25.Feb.2025

Reporting Summary

Nature Portfolio wishes to improve the reproducibility of the work that we publish. This form provides structure for consistency and transparency in reporting. For further information on Nature Portfolio policies, see our Editorial Policies and the Editorial Policy Checklist.

Statistics

For all statistical analyses, confirm that the following items are present in the figure legend, table legend, main text, or Methods section.

n/a	Confirmed	
☐	☒	The exact sample size (*n*) for each experimental group/condition, given as a discrete number and unit of measurement
☐	☒	A statement on whether measurements were taken from distinct samples or whether the same sample was measured repeatedly
☒	☐	The statistical test(s) used AND whether they are one- or two-sided<br>*Only common tests should be described solely by name; describe more complex techniques in the Methods section.*
☒	☐	A description of all covariates tested
☒	☐	A description of any assumptions or corrections, such as tests of normality and adjustment for multiple comparisons
☐	☒	A full description of the statistical parameters including central tendency (e.g. means) or other basic estimates (e.g. regression coefficient) AND variation (e.g. standard deviation) or associated estimates of uncertainty (e.g. confidence intervals)
☒	☐	For null hypothesis testing, the test statistic (e.g. *F*, *t*, *r*) with confidence intervals, effect sizes, degrees of freedom and *P* value noted<br>*Give P values as exact values whenever suitable.*
☒	☐	For Bayesian analysis, information on the choice of priors and Markov chain Monte Carlo settings
☒	☐	For hierarchical and complex designs, identification of the appropriate level for tests and full reporting of outcomes
☒	☐	Estimates of effect sizes (e.g. Cohen's *d*, Pearson's *r*), indicating how they were calculated

Our web collection on statistics for biologists contains articles on many of the points above.

Software and code

Policy information about availability of computer code

Data collection	AFM: JPK Nanowizard Control; QCM-D: QSoft; SPR: BioEval
Data analysis	AFM: JPK Data Processing (AFM force curve analysis), and OriginLab Pro (further statistical analysis and graphing);<br>QCM-D: QTools (primary data analysis), and OriginLab Pro (graphing); SPR: OriginLab Pro (further analysis and graphing)

For manuscripts utilizing custom algorithms or software that are central to the research but not yet described in published literature, software must be made available to editors and reviewers. We strongly encourage code deposition in a community repository (e.g. GitHub). See the Nature Portfolio guidelines for submitting code & software for further information.

Data

Policy information about availability of data

All manuscripts must include a data availability statement. This statement should provide the following information, where applicable:

- Accession codes, unique identifiers, or web links for publicly available datasets
- A description of any restrictions on data availability
- For clinical datasets or third party data, please ensure that the statement adheres to our policy

Data availability statement.
The data that support this study are available from the corresponding author, David G. Jackson, upon request. Plasmids generated in this study will be made

available on request. The supporting computational data are provided via public repository 87. The crystal structures generated in this study have been deposited in the RCSB Protein Data Bank database under accession codes 8ORX [https://doi.org/10.2210/pdb8ORX/pdb] for mLYVE-1 Apo, 8OS2 [https://doi.org/10.2210/pdb8OS2/pdb] for hLYVE-1 Apo, 8OX3 [https://doi.org/10.2210/pdb8ox3/pdb] for mLYVE-1 with HA8, and 8OXD [https://doi.org/10.2210/pdb8OXD/pdb] for mLYVE-1 with HA10. The DFS, QCM/D and MD data generated in this study are provided in the accompanying Source Data files and supporting computational data are provided via public repository 87. All other unique/stable reagents generated in this study are available from the lead contact with a completed Materials Transfer Agreement.

Research involving human participants, their data, or biological material

Policy information about studies with human participants or human data. See also policy information about sex, gender (identity/presentation), and sexual orientation and race, ethnicity and racism.

Reporting on sex and gender	The manuscript does not involve human participants
Reporting on race, ethnicity, or other socially relevant groupings	The manuscript does not involve human participants
Population characteristics	The manuscript does not involve human participants or populations
Recruitment	The manuscript does not involve human participants
Ethics oversight	The manuscript does not involve human participants, so no ethics issues are involved

Note that full information on the approval of the study protocol must also be provided in the manuscript.

Field-specific reporting

Please select the one below that is the best fit for your research. If you are not sure, read the appropriate sections before making your selection.

☒ Life sciences ☐ Behavioural & social sciences ☐ Ecological, evolutionary & environmental sciences

For a reference copy of the document with all sections, see nature.com/documents/nr-reporting-summary-flat.pdf

Life sciences study design

All studies must disclose on these points even when the disclosure is negative.

Sample size	AFM force curves: At least 50 force curves were acquired if the interactions did not exhibit stochastic variations (i.e., for LYVE-1 on HA in tail configuration) and several hundred to thousand individual force curves were acquired if the interactions exhibited stochastic variations (i.e., for CD44 on HA in tail configuration, and for LYVE-1 on HA in loop configuration).<br>QCM-D: Not applicable. SPR: Not applicable.
Data exclusions	AFM: Only rupture events appearing at tip-substrate distances larger than 250 nm were considered for further analysis (see Methods for details).<br>QCM-D: No data points were excluded. SPR: No data points were excluded.
Replication	AFM: All experiments were performed at least twice with distinct yet identically prepared AFM probes and planar substrates.<br>QCM-D: All experiments were carried out in duplicate. SPR: All experiments were carried out in triplicate.
Randomization	AFM: Not applicable. QCM-D: Not applicable. SPR: Not applicable.
Blinding	AFM: Not applicable. QCM-D: Not applicable. SPR: Not applicable.

Reporting for specific materials, systems and methods

We require information from authors about some types of materials, experimental systems and methods used in many studies. Here, indicate whether each material, system or method listed is relevant to your study. If you are not sure if a list item applies to your research, read the appropriate section before selecting a response.

Materials & experimental systems

n/a	Involved in the study	
☐	☒	Antibodies
☐	☒	Eukaryotic cell lines
☒	☐	Palaeontology and archaeology
☒	☐	Animals and other organisms
☒	☐	Clinical data
☒	☐	Dual use research of concern
☒	☐	Plants

Methods

n/a	Involved in the study	
☒	☐	ChIP-seq
☒	☐	Flow cytometry
☒	☐	MRI-based neuroimaging

Antibodies

Antibodies used: Mouse anti-human LYVE-1 mAb clone 3A (derived in house and referenced in text)
Anti-human LYVE-1 mAb 8C (derived in house and referenced in text)

Validation: Validation is published in the references cited in the text

Eukaryotic cell lines

Policy information about cell lines and Sex and Gender in Research

Cell line source(s): HEK 293T from Cancer Research UK and ATCC: CHO-K1 (D28-W1) from Lonza Biologics

Authentication: None was further authenticated

Mycoplasma contamination: The cell lines routinely tested negative for mycoplasma

Commonly misidentified lines (See ICLAC register): Neither of the cell lines used is listed in the CLAC register, meaning they are not commonly mis-identified

Plants

Seed stocks: The study did not use any plants

Novel plant genotypes: The study did not use any clinical data

Authentication: The study did not use any clinical data
